# Inborn errors of immunity: Manifestation, treatment, and outcome—an ESID registry 1994–2024 report on 30,628 patients

**DOI:** 10.70962/jhi.20250007

**Published:** 2025-07-17

**Authors:** Gerhard Kindle, Mickaël Alligon, Michael H. Albert, Matthew Buckland, J. David Edgar, Benjamin Gathmann, Sujal Ghosh, Antonios Gkantaras, Alexandra Nieters, Claudio Pignata, Peter N. Robinson, Stephan Rusch, Catharina Schuetz, Svetlana Sharapova, Benjamin Shillitoe, Fabio Candotti, Andrew J. Cant, Jean-Laurent Casanova, Amos Etzioni, Alain Fischer, Isabelle Meyts, Luigi D. Notarangelo, Martine Pergent, C.I. Edvard Smith, ESID Registry Working Party, Lennart Hammarström, Bodo Grimbacher, Mikko R.J. Seppänen, Nizar Mahlaoui, Stephan Ehl, Markus G. Seidel

**Affiliations:** 1 Institute for Immunodeficiency, Center for Chronic Immunodeficiency, Medical Center-University of Freiburg, Faculty of Medicine, University of Freiburg, Freiburg, Germany; 2 Centre for Biobanking FREEZE, Medical Center-University of Freiburg, Faculty of Medicine, University of Freiburg, Freiburg, Germany; 3 French National Reference Center for Primary Immune Deficiencies, Necker Enfants Malades University Hospital, Assistance Publique-Hôpitaux de Paris, Paris, France; 4 Department of Pediatrics, Dr. von Hauner Children’s Hospital, Ludwig-Maximilians Universität München, Munich, Germany; 5 Barts Health National Health Service Trust, London, UK; 6 Molecular and Cellular Immunology Section, Immunity and Inflammation Department, Great Ormond Street Institute of Child Health, London, UK; 7 Department of Immunology, St James’s Hospital, and School of Medicine, Trinity College Dublin, Dublin, Ireland; 8 Department of Pediatric Oncology, Hematology and Clinical Immunology, Medical Faculty, Center of Child and Adolescent Health, Heinrich-Heine-University and University Hospital, Duesseldorf, Germany; 9 Pediatric Immunology and Rheumatology Referral Center, 1st Department of Pediatrics, Aristotle University of Thessaloniki, Hippokration General Hospital, Thessaloniki, Greece; 10 Department of Translational Medical Sciences, Federico II University, Naples, Italy; 11 Berlin Institute of Health, Charité - Universitätsmedizin Berlin, Berlin, Germany; 12 The Jackson Laboratory for Genomic Medicine, Farmington, CT, USA; 13 Department of Pediatrics and University Center for Rare Diseases, Medizinische Fakultät Carl Gustav Carus, Technische Universität, Dresden, Germany; 14 German Center for Child and Adolescent Health, Partner Site Leipzig/Dresden, Dresden, Germany; 15 Research Department, Belarusian Research Center for Pediatric Oncology, Hematology and Immunology, Minsk Region, Belarus; 16 Paediatric Immunology and Infectious Diseases, Sheffield Children’s NHS Foundation Trust, Sheffield, UK; 17 Division of Immunology and Allergy, Lausanne University Hospital and University of Lausanne, Lausanne, Switzerland; 18 Translational and Clinical Research Institute, Newcastle University, Newcastle upon Tyne, England; 19 Department of Paediatric Immunology and Infectious Diseases, Great North Children’s Hospital, Newcastle upon Tyne, England; 20 Laboratory of Human Genetics of Infectious Diseases, INSERM U1163, Necker Hospital for Sick Children, Paris, France; 21 Imagine Institute, University Paris Cité, Paris, France; 22 St. Giles Laboratory of Human Genetics of Infectious Diseases, Rockefeller Branch, The Rockefeller University, New York, NY, USA; 23 Howard Hughes Medical Institute, New York, NY, USA; 24 Department of Pediatrics, Necker Hospital for Sick Children, Assistance Publique-Hôpitaux de Paris, Paris, France; 25 Faculty of Medicine, Technion, Haifa, Israel; 26 Unité d’immunologie Pédiatrique, Hôpital Necker Enfants Malades, Paris, France; 27 Department of Pediatrics, Department of Microbiology, Immunology and Transplantation, ERN-RITA Core Center, University Hospitals Leuven, KU Leuven, Leuven, Belgium; 28 Laboratory of Clinical Immunology and Microbiology, National Institute of Allergy and Infectious Diseases, National Institutes of Health, Bethesda, MD, USA; 29 International Patient Organisation for Primary Immunodeficiencies, Brussels, Belgium; 30 Department of Laboratory Medicine, Clinical Immunology, Karolinska Institutet, Stockholm, Sweden; 31 Department of Infectious Diseases, Karolinska University Hospital, Stockholm, Sweden; 32 Karolinska ATMP Center, Karolinska Institutet, Karolinska University Hospital, Stockholm, Sweden; 33 Division of Immunology, Department of Medical Biochemistry and Biophysics, Karolinska Institutet, Stockholm, Sweden; 34 Clinic of Rheumatology and Clinical Immunology, Center for Chronic Immunodeficiency, Medical Center, Faculty of Medicine, Albert-Ludwigs-University of Freiburg, Freiburg, Germany; 35 German Center for Infection Research, Satellite Center Freiburg, Freiburg, Germany; 36 Centre for Integrative Biological Signalling Studies, Albert-Ludwigs University, Freiburg, Germany; 37 RESIST - Cluster of Excellence 2155 to Hanover Medical School, Satellite Center Freiburg, Freiburg, Germany; 38 Rare Disease Center and Pediatric Research Center, Children and Adolescents, University of Helsinki, HUS Helsinki University Hospital, ERN-RITA Core Center, RITAFIN, Helsinki, Finland; 39 Translational Immunology Research Program, University of Helsinki, Helsinki, Finland; 40 Pediatric Immuno-Hematology and Rheumatology Unit, Necker Enfants Malades University Hospital, Assistance Publique-Hôpitaux de Paris, Paris, France; 41 Division of Pediatric Hematology and Oncology, Department of Pediatric and Adolescent Medicine, Styrian Children’s Cancer Research Unit for Cancer and Inborn Errors of the Blood and Immunity in Children, Medical University of Graz, Graz, Austria.

## Abstract

The European Society for Immunodeficiencies patient registry (ESID-R), established in 1994, is one of the world’s largest databases on inborn errors of immunity (IEI). IEI are genetic disorders predisposing patients to infections, autoimmunity, inflammation, allergies, and malignancies. Treatments include antimicrobial therapy, immunoglobulin replacement, immune modulation, stem cell transplantation, and gene therapy. Data from 194 centers in 33 countries capture clinical manifestations and treatments from birth onward, with annually expected updates. This report reviews the ESID-R’s structure, data content, and impact. The registry includes 30,628 patient datasets (aged 0–97.9 years; median follow-up: 7.2 years; total 825,568.2 patient-years), with 13,550 cases in 15 sub-studies. It has produced 84 peer-reviewed publications (mean citation rate: 95). Findings include real-world observations of IEI diagnoses, genetic causes, clinical manifestations, treatments, and survival trends. The ESID-R fosters global collaboration, advancing IEI research and patient care. This report highlights the key role of the multinational ESID-R, led by an independent medical society, in evidence-based discovery.

## Introduction

Inborn errors of immunity (IEI) are genetic disorders affecting immunity. They almost invariably increase the susceptibility to infections and may cause autoimmunity, inflammation, allergy, and predispose individuals to malignancy (1, 2, 3, 4, 5, 6). IEI can manifest at any age and are characterized by a spectrum of symptoms related to impaired or uncontrolled immune responses. These often have a serious impact on the health and quality of life of those affected. Many IEI are rare diseases, including ultra-rare and hyper-rare forms (i.e., frequently <<1/2,000 persons affected). Thus, an international strategy was needed to collect a meaningful number of datapoints for cross-sectional analyses and longitudinal clinical observations of natural courses of specific IEI.

The European Society for Immunodeficiencies registry (ESID-R) was founded in 1994 by ESID, a nonprofit medical specialist society, creating a central database for primary immunodeficiencies. These are currently referred to as IEI or primary immune disorders (PID), differentiating them from secondary immune disorders (7). The ESID-R mainly serves to contain, store, and enable the analysis of IEI/PID data to improve the understanding of these diseases and their underlying immunobiology. Historically, the ESID-R was operated for the first 10 years as a hard copy-based database, and data were submitted by fax to the first chairs of the ESID-R, Lennart Hammarström and Mohammad Abedi, in Huddinge, Sweden. From 2000 to 2004, Bodo Grimbacher with coworkers in Freiburg, Germany, developed and implemented the first web-based version of the ESID-R (8, 9). In 2014, another major revision was carried out, headed by Stephan Ehl, Freiburg. The two key drivers of this second redesign were the goal to allow registry participation at three different levels according to center resources and the need to improve data quality so that a high level of confidence in the accuracy of the data could be inferred for clinical application and publication. In addition to inbuilt quality checks, the migration of existing data to the third version of the ESID-R and the entry of new patient data since then required a manual validation process of all included patients lacking a genetic diagnosis to meet the simultaneously developed clinical-working criteria for IEI/PID diagnoses, compiled and published by a group of experts (10). In late 2024, ESID decided to move the ESID-R technical and physical foundation from the Medical Center of the University of Freiburg to a commercial clinical trials operator (Castor-edc, Netherlands) to improve data, system, and access security. Maintaining the three-level study structure and the mandatory diagnosis validation process, this currently new, fourth version of the ESID-R is expected to facilitate the generation of data modules by research groups, allowing decentralized sub-study (electronic case report form) programming, independent data exports for center or sub-study analyses, automated center dashboards, and add-on features such as patient reporting.

Here, we present major findings from clinical observations of 30,628 patients, based on the current ESID-R dataset. These findings indicate epidemiologically relevant disease distributions and the calculated prevalence of diagnoses in registered patients, their clinical manifestations, diagnostic delays, treatment, disease course, and survival probabilities across all IEI/PID categories. Furthermore, we describe the organizational and technical evolution of the ESID-R, its relationship with the international registry landscape, and its role as a research (sub-)study platform. These data are of the utmost relevance to anyone affected by immune disorders or involved in patient care, management and therapy, and drug and policy development for patients with IEI/PID around the world.

## Results

On the end date chosen for inclusion in March 2024, the ESID-R contained data for 30,628 IEI/PID patients from 194 participating centers in 33 European and other, mostly neighboring, countries. There was a steady increase in the registration of patients over time (Fig. S1 A), apart from a temporary decline in patient numbers and, to a lesser extent, in center numbers (Fig. S1 B) shortly after 2014, due to structural platform changes in 2014. These required centers to verify their patients to improve the accuracy of existing data. This allowed us to derive patient distribution by country and the minimal prevalence of PID/IEI (Table S1 and Fig. S2).

### Patient characteristics

**Figure 1.**
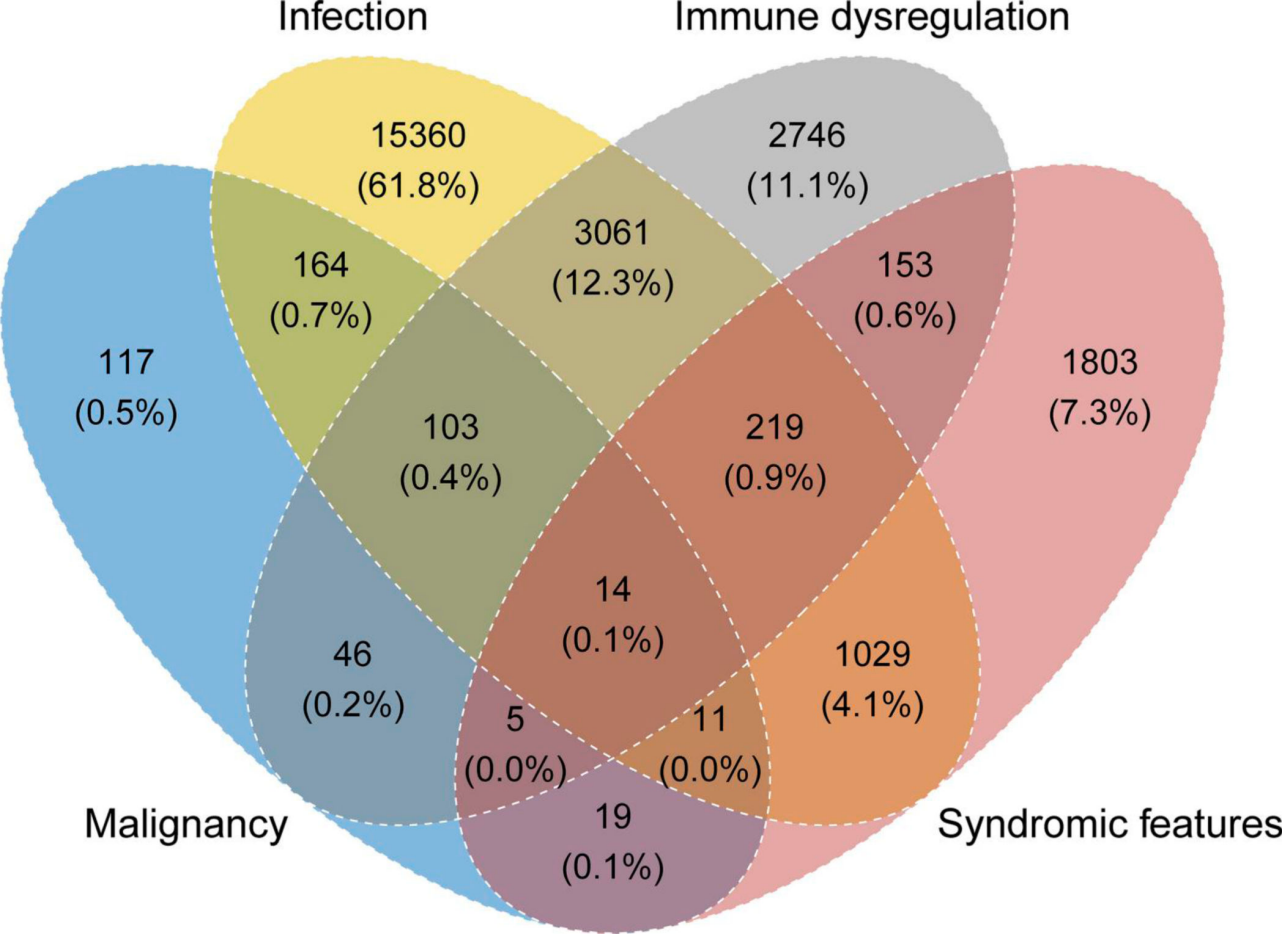
Main manifestations at onset of IEI or PID. Venn diagram of main manifestations of IEI/PID with absolute patient numbers and proportions.

Fig. 1 shows the clinical manifestations that led to IEI/PID diagnosis. Ages at onset, clinical, and genetic diagnosis are shown in Fig. S1 C. A steady decline in new diagnoses was observed with age, although a small second peak and plateau were seen in adulthood, and a trend for earlier identification of genetic diagnoses was observed. The delay from the onset of symptoms to clinical diagnosis and between clinical and genetic diagnosis is shown by year of clinical or genetic diagnosis, i.e., the endpoint of each delay, over the last 20 years in Fig. S1 D. As reported previously (11), the majority of 80.3% of patients have infections on their way to diagnosis, but only in 61.8% as sole recorded manifestation of IEI, followed by features of immune dysregulation in 11.1% and syndromic manifestations in 7.3% as sole clinical presentation at diagnosis (*n* = 15,360; 2,746; 1,803, respectively; Fig. 1). Malignancies were among the first manifestations of IEI in 479 patients and the only initial manifestation in 117 patients (0.5%; Fig. 1).

Table 1 shows the main patient demographic and diagnosis categories, comorbidities such as malignancy, COVID-19, and living status (Fig. S3). Fig. S4 shows the patient numbers and their distribution according to their International Union of Immunological Societies (IUIS) classification, disease name, and genetic diagnosis (see detailed interactive version at https://esid.org/html-pages/Suppl%20Fig%203_ESID_30k_sunburst_PID.html). About half of all patients suffer from primary antibody deficiencies (PAD; *n* = 15,123), followed in descending order by combined immunodeficiencies (CID) with syndromic features (“syndromic”; *n* = 4,239), phagocytic disorders (“phagocyte”; *n* = 2,548), CID (*n* = 2,531), and primary diseases of immune dysregulation (PIRD; *n* = 2,171; Table 1 and Fig. S4). Table 1 presents the ages at onset, clinical diagnosis, genetic confirmation, last follow-up, and the diagnostic delay. The proportion of patients reported to have suffered from malignancies was 8.9% (*n* = 1,783). Malignancies were reported as occurring in all subgroups of patients with IEI/PID with moderately varying frequencies, corroborating the notion that IEI/PID are cancer predisposition syndromes. The proportion of patients reported to have been affected by COVID-19 was highest in PAD (35.9%; total cohort: 29.7%). More than half of CID patients received curative therapy (52.6%), followed by those with bone marrow failure syndromes (BMF; 39.8%), PIRD, and phagocyte disorders (30.3% and 25.8%, respectively). Table S2 shows the causes of death.

### Representation of genetic IEI diagnoses

**Figure 2.**
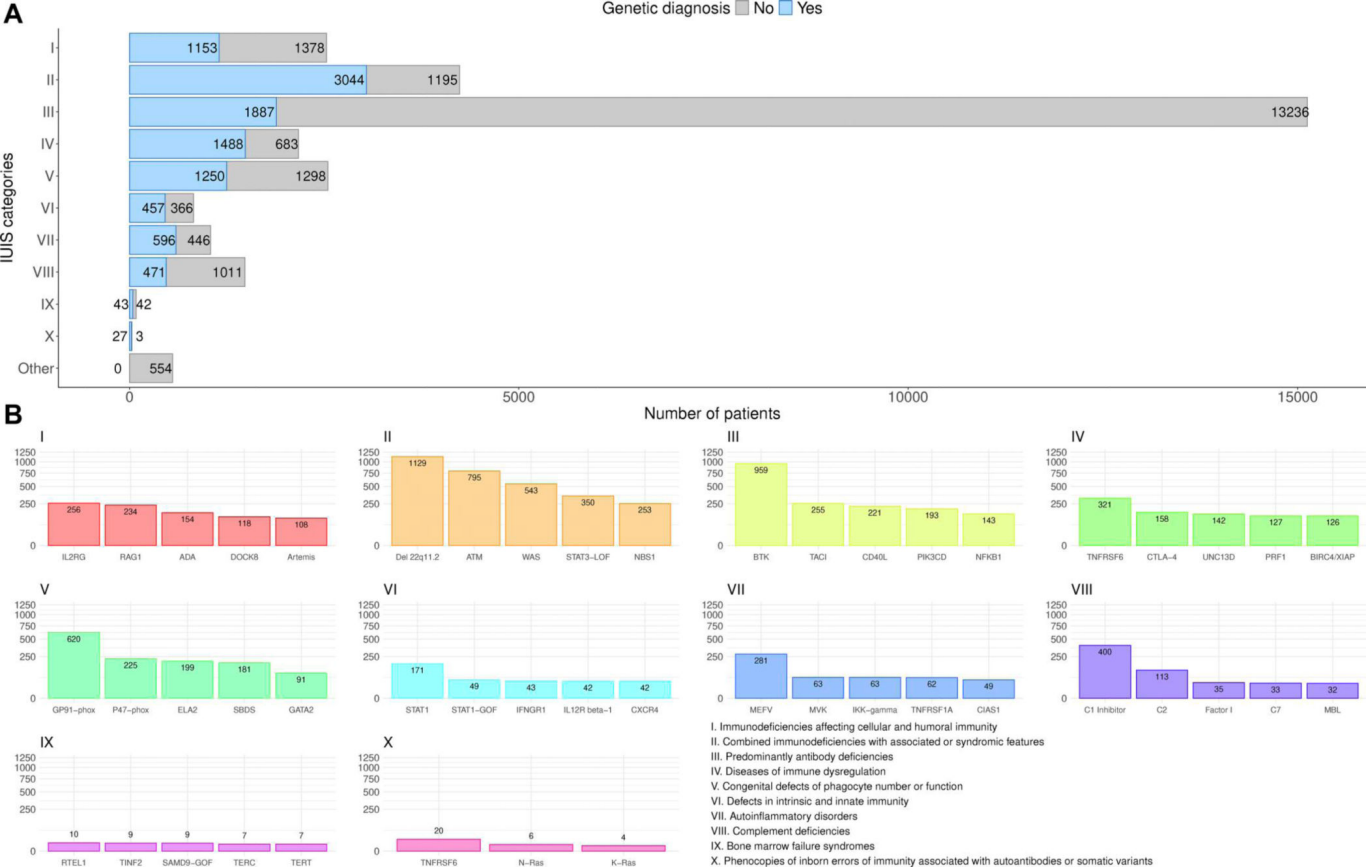
Representation of genetic diagnoses of known IEI/PID in the ESID-R. **(A, upper panel)** The number and proportion of patients with a genetic versus those without a genetic diagnosis is shown in descending order. **(B, lower panel)** The top 5 genetic defects or deletions registered in the ESID-R per all 10 IUIS categories of IEI are presented; the nomenclature in registry diagnosis and gene fields was not regularly updated/changed, showing, e.g., GP91-phox instead of *CYBB* and p47-phox instead of *NCF1*.

Fig. 2 shows the absolute numbers of patients with a documented genetic diagnosis (*n* = 12,774, 44.5%) versus those without (*n* = 15,905, 55.5%, including patients not genetically tested) by IEI category. As expected, within the largest patient subgroup (PAD), the genetic diagnosis is lacking for the majority of patients (Fig. 2 A), and the clinical diagnosis of common variable immunodeficiency (CVID) is attributed, whereas patients with CID with syndromic features had the highest proportion of genetic diagnoses. The top five genes mutated per IEI category are shown in Fig. 2 B, with *IL2RG* being the most frequently reported germline genetic cause of CID; *22q11.2* deletion syndrome, of CID with syndromic features; *BTK*, of PAD; *TNFRSF6*, of category IV, diseases of immune dysregulation; *CYBB*, of phagocyte disorders; *STAT1*, of intrinsic or innate immune disorders; *MEFV*, of autoinflammatory syndromes; C1 inhibitor, of complement deficiencies; *RTEL*, of BMF; and somatic *TNFRSF6*, of phenocopies. The top 50 genetic causes of IEI from the ESID-R are shown with patient numbers in descending order in Fig. S5.

### Treatment modalities according to IEI categories

**Figure 3.**
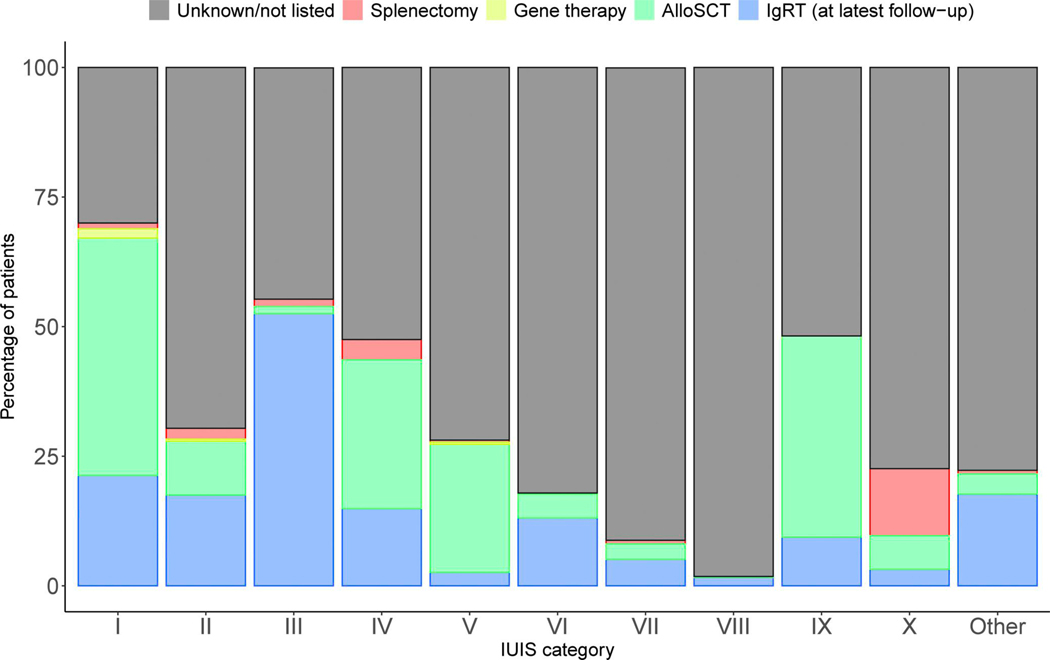
Major treatment modalities of patients with IEI/PID as recorded in the ESID-R. Relative proportions of major treatments such as IGRT (data on route, interval, and dose were recorded but are not shown), allogeneic HSCT (AlloSCT), autologous GT, and splenectomy. Only a proportion of centers recorded data on immune-modifying treatment (not shown), and the ESID-R does not capture data on antimicrobial therapy. “Unknown/not listed” and IGRT refer to the latest follow-up time point.

Immunoglobulin replacement therapy (IGRT), hematopoietic stem cell transplantation (HSCT), gene therapy (GT), and splenectomy are recorded in the ESID-R and listed in Table S3. As expected, the highest number and proportion of IGRT-receiving patients is seen in the subgroup of patients with PAD (Fig. 3). The highest absolute numbers of HSCT procedures were performed in patients with CID, followed by those with PIRD and phagocytic disorders. IGRT and HSCT were documented in patients with IEI/PID in any category. GT, an evolving curative treatment option devoid of some risks associated with HSCT, such as alloreactivity, was recorded in a descending order for CID, syndromic, and phagocyte disorders (Fig. 3). Splenectomy was recorded relatively frequently in patients with phenocopies, but it was also documented for any IEI category. The use of immune-modulatory treatments, such as anti-inflammatory, cell-depleting, or pathway-directed (targeted) therapies, and the rare cases of thymus or solid organ transplantation were recorded but are not shown here, as the heterogeneity of the data exceeds the scope of this general report.

### Survival probabilities of patients with IEI

**Figure 4.**
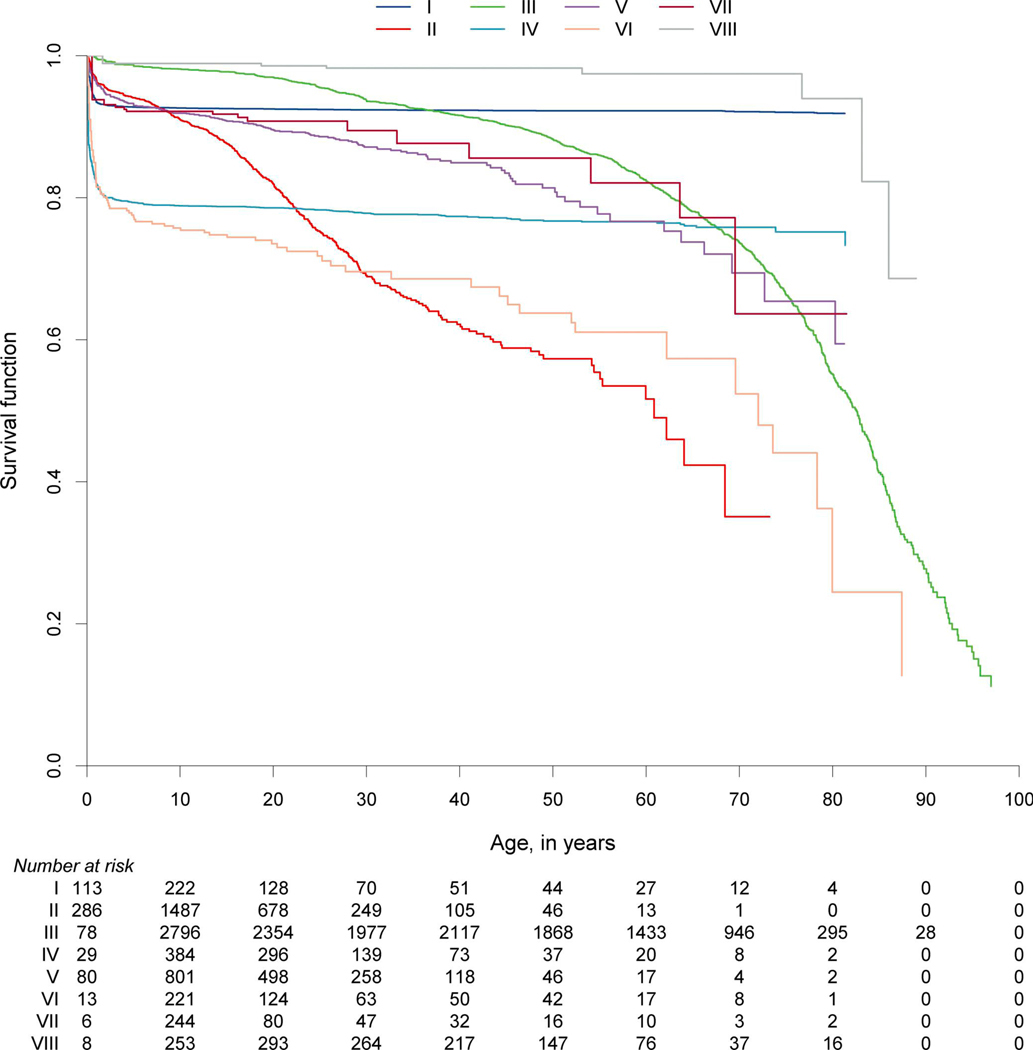
Survival probabilities of main categories of IEI/PID and the living status at last news. Inverse cumulative incidence curves as described and referenced in the Supplemental material. Start = age at diagnosis, stop = age at last news, and event = living status (0 = censored, 1 = deceased first, and 2 = curative therapy first). There were 21,206 patients censored; 1,960 patients who deceased first; and 2,901 patients who had a curative therapy first (not showing deaths after curative therapy); roman numbers refer to the IUIS categories for IEI/PID as listed in Fig. 2 B.

We hypothesized that the survival probabilities of patients in the 10 IUIS categories of IEI differed due to their varying predispositions to life-threatening infections, malignancies, autoimmunity, or other manifestations and complications associated with their underlying conditions. Although the data granularity in the entire ESID-R with respect to patient follow-up intervals is not comparable with that in disease-specific prospective cohort studies, we could plot the reversed cumulative incidence function, due to the presence of competing risks, based on the relatively large patient numbers in each IEI category (Fig. 4; see Fig. S6 for the same curves with confidence intervals). Of note, natural biases such as underreporting of patients who died from IEI before diagnosis or of patients with a mild phenotype exist, and numbers at risk increased over the first few years of the observation period (0–97.9 years of age) due to the later time points of inclusion or diagnosis. We detected a steep early decline in survival in many IE categories while curves plateaued, (1) methodologically, in some where definitive treatments exist, or (2) in genotypes with variable penetrance (e.g., CID and PIRD). Additionally, while patients with PAD showed a continuous decline in survival probability from a young age across all age groups, the decline in the survival of patients with phagocyte and innate immune disorders showed an initial drop. This finding suggests that a proportion of patients are at very high risk during their first 5 years of life. Diagnoses of early deceased patients with PIRD (*n* = 70 under 5 years of age) were mostly due to disorders with a high risk of hemophagocytic lymphohistiocytosis (81.4%); premature deaths in “innate” IEI were frequently due to IRAK4 or MyD88 deficiencies (Table S4). Patients with IEI/PID with syndromic features had a triphasic survival probability. After observing an initial decline in the first 4 years of life, we detected a second pronounced decline in survival probability in the patients’ second and third decades of life, most likely due to the increased risk of malignancies in many patients in this subgroup (>25%, see Table S2), and a third, relatively steep decline in patients in their sixth to seventh decades.

### The ESID-R in the international data source landscape

Other continental or cross-regional IEI/PID registries of varying temporal and geographical depth include the global Jeffrey Modell Foundation Centers Network (>94,000 patients), the United States Immunodeficiency Network registry in the USA (>5,000), the Latin American Society for Immunodeficiencies registry in Latin America (>9,000), the Australian AusPIPS registry (>1,500) as part of the Australasian network, Japanese Society for Immunodeficiency and Autoinflammatory Diseases (>1,200) in Japan as part of the Asia-Pacific (Asia-Pacific Society for Immunodeficiencies) network, the Canadian registry (Canadian IEI National Registry) founded in 2024, and the registries of the Primary Immune Deficiency Treatment Consortium (PIDTC) of North America; in addition, many national registries exist inside or outside of ESID (12). Those reported in Europe are shown in Fig. S7 and Table S5. Furthermore, the ESID-R is listed as official data source in catalogues of the European Medical Agency and of European Rare Disease Registry Infrastructure, the European Union (EU) rare disease platform, which are metadata repositories to increase visibility and facilitate the use of rare disease patients’ data.

### Scientific impact of the ESID-R and sub-studies

**Figure 5.**
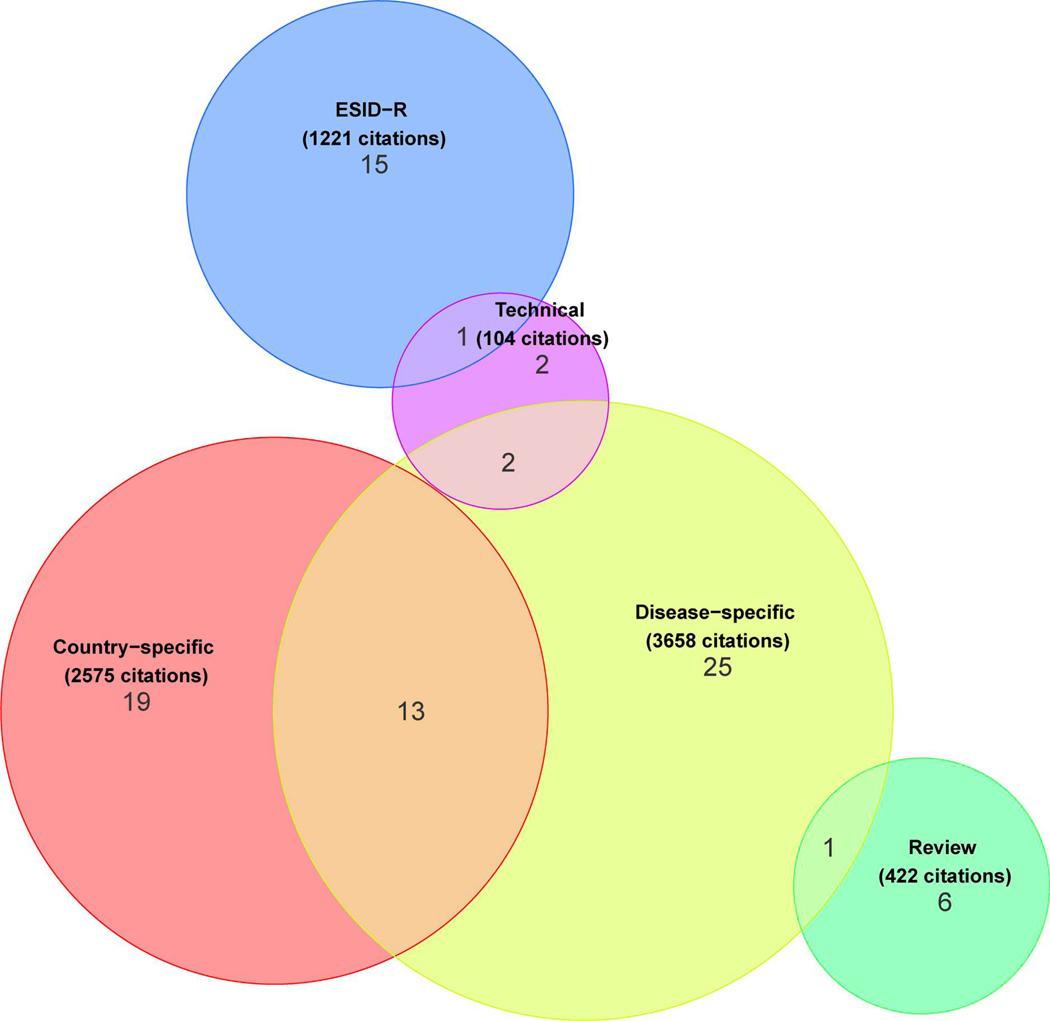
Number, categories, and citation counts of ESID-R–based publications. A total of 7,980 citations of 84 peer-reviewed ESID-R–based publications until early 2024 were recorded. See also the website for regular updates of ESID-R–related publications at https://esid.org/working-parties/registry-working-party/registry-publications/.

Up to the end date chosen for data inclusion in this manuscript, 84 peer-reviewed publications resulted from projects deriving data directly from the ESID-R (Fig. 5 and ESID website [13]). We evaluated this scientific output by categorizing and counting the publications and their citations as follows: disease-specific natural history studies (*n* = 25; 3,658 citations), country-specific epidemiological studies (*n* = 19; 2,575 citations), six reviews (422 citations), two on technical aspects (104 citations), and 15 of our own registry-conducted studies (e.g., on first manifestations or on working definitions for the clinical diagnosis of IEI/PID, 1,221 citations), plus 17 studies that could have been assigned to multiple or overlapping categories (Fig. 5), resulting in a mean citation rate of 95 per publication.

## Discussion

The ESID-R is a large and growing real-world database enabling powerful analyses that have continuously generated epidemiological and disease-specific observational results since 1994. It contributes to knowledge and improvements in patient care in IEI in Europe and beyond. The present analysis illustrates the current distribution of (1) IEI diagnoses recorded in contributing centers and countries; (2) major treatment modalities between all IEI categories; (3), ages at onset, at clinical, and at genetic diagnosis; and (4) survival probabilities. Our results underline the recommendation to potentially implement newborn screening (NBS) for certain additional IEI other than severe CID (SCID). The 7,980 citations of 84 peer-reviewed ESID-R–based publications as of early 2024 demonstrate the success of the ESID-R as a platform for research sub-studies and their substantial scientific impact. Hence, the ESID-R provides a clear example of how data collection and collaboration can benefit patients with rare diseases.

### Survival probabilities, early diagnosis, and NBS

NBS for SCID by measuring T cell receptor with or without kappa-deleting recombination excision circles in dried blood spots has been implemented in many countries around the world (14, 15, 16). A substantial survival benefit for patients diagnosed and treated by early HSCT was demonstrated and recently confirmed in a longitudinal study of the PIDTC (17). The steep early decline in survival we observed in patients with disorders of immune regulation and innate IEI may argue for an extension of NBS to other immediately life-threatening IEI (e.g., familial hemophagocytic lymphohistiocytosis [FHL], XLP1, MyD88/IRAK4 deficiencies, and IPEX syndromes). To extend the spectrum of early IEI–NBS, current techniques may be supplemented by RNA sequencing or germline genetic testing, such as targeted sequencing panels and whole exome or genome sequencing. Feasibility studies of such extended NBS are ongoing in regional or national programs (18). However, the vast ethical and social implications of any omics-based addition like baby genome screening will require careful consideration of the risks and benefits (19).

### Methodological and organizational limitations

Physicians and other documentarists regularly collect data for the ESID-R on a voluntary basis, with data quality and quantity (i.e., data depth and accuracy) varying substantially between centers and across countries. Depending on resources dedicated to data documentation, an underestimation of the prevalence of IEI/PID, assumed to be widely similar across Europe, of around 30% was demonstrated (20). While all centers can participate in the ESID-R and scientific sub-studies for free, minimum infrastructure is required. With the rare exception of per-patient fees in pharma-sponsored level-3 studies, ESID does not financially reimburse for data entry. The participants mainly benefit from the international academic collaboration and representation, which increases their awareness for disease phenotypes and current clinical research questions. Also, each center may obtain their own (local and regional) epidemiological data within a legally and ethically approved and financially sustainable technical framework (see survey results in the supplemental material). Furthermore, ESID has no means of monitoring the data quality other than by inbuilt checks for logic and completeness at the time point of data entry. So far, automated transfers of data from electronic health records (eHR) into the ESID-R, which have the potential to overcome many of the aforementioned obstacles and to enhance both the quality and quantity of recorded data, have not been attempted due to regulatory obstacles and the heterogeneity of eHRs in the 194 participating centers. All of the above, along with the fact that some monogenic IEIs have only been described recently while others have been recognized for decades, introduces a systemic ascertainment bias into the dataset when comparing, e.g., numbers of diagnoses and time of survival. For example, X-linked agammaglobulinemia (caused by mutations in *BTK*) (21) is probably not eight times more prevalent than, e.g., the autosomal-dominant CTLA4-(haplo)insufficiency (22, 23) or NFKB1-(haplo)insufficiency (24). In summary, we build on the trust of participants and the success of this huge cooperative effort to continue the ESID-R in its fourth technical version starting from late 2024, serving as an up-to-date, simple, and free platform for inherently motivated international scientific clinical research collaboration.

### Challenges of reporting genetic data

Collecting data from the heterogenous patient population of individuals with IEI/PID and storing these in a registry presents many challenges. Scientists estimate that current routine next-generation sequencing technologies can reveal a molecular cause in ∼30–35% of IEI cases (18). It is noteworthy that information classifying detected variants by applying American College of Medical Genetics criteria or performing functional validation is not collected in the ESID-R. Phenotype information derived from the ESID-R might support the efforts of the ClinGen Immunology Gene and Variant Curation Expert Panels (25). The incorporation of genetic data beyond known pathogenic mutations, ideally linked to precisely defined phenotypes according to the human phenotype ontology (HPO) (26), represents a biologically interesting future challenge. This includes the verification of variants of unknown significance, intronic changes, somatic mutations and mosaicism, epigenetic factors, variable penetrance, and other less-studied factors such as noncoding modifier alleles and monoallelic expression (27).

### Link to other registries, synergies, overlapping efforts

From a scientific perspective, it has become increasingly important to compare outcomes for IEI/PID patients with and without cellular therapy (HSCT or GT). The registry of the European Society for Blood and Marrow Transplantation (EBMT) currently registers definitive cellular therapies in about 700 patients with IEI per year (28), many of whom are also registered in the ESID-R. Hence, to optimize future research, datasets from the ESID and the EBMT registries for the same patients should be combined. Better alignment of the registries and their data fields is urgently needed to facilitate such studies, and we expect the new technical platform to facilitate data exports and imports alike across providers. Furthermore, the IUIS classification has gradually incorporated more diseases from overlapping specialties (e.g., hematology-oncology, rheumatology, and gastroenterology), many of which are (also) covered by other medical societies and patient registries. Thus, although data from many of these patients are stored in the ESID-R, their proportion in the ESID-R does not reflect the real-world distribution. The registry of the European Reference Network for Rare Immunological and Autoimmune Diseases, an EU sponsored network of healthcare providers from reference centers for PID/IEI, rheumatology, autoinflammatory, and autoimmune diseases, aims to collect common data elements from patients across these disease areas. This initiative may help to avoid redundancy. Finally, using independent (meta-)identifiers such as the European SPIDER-ID (29) in all registries would help scientists to disentangle the registry landscape.

### Future: Artificial intelligence (AI) in registry work for data analysis and interpretation

Advanced software tools and AI (e.g., large language models, natural language processing, and machine learning) will certainly transform clinical decision-making and other crucial processes in medicine, including the field of IEI (30, 31) Automated eHR-scanning and data-harvesting processes, ideally for specific terms in accordance with HPO, medical reports, and disease classification codes (e.g., ICD-11 or ORPHA codes), but also for free text may be used to identify currently undiagnosed IEI/PID patients. These individuals might benefit from early screening by preventing complications. Accordingly, as a first step, data from an IEI/PID registry like the ESID-R that includes data from patients with an established diagnosis could be used to train AI models and to predict a monogenic IEI or at least the most likely affected pathway in patients who lack a genetic diagnosis. Consequently, patients may undergo screening for disease-specific risks and receive appropriate therapy early in their course. In addition to AI-assisted automated data extraction from eHRs to feed patient registries with structured information, complementing the ESID-R with AI-based tools has the potential to transform this data-collection platform to an electronic IEI/PID patient management assistant in the future.

### Conclusions and perspectives

The rarity and complexity of many IEI grants them an orphan status regarding pharmaceutical research and development. The feasibility of clinical research, including drug trials and post-authorization efficacy and safety studies, thus depends on large networks of academic institutions and medical specialist societies. As one of the largest registries, containing extensive longitudinal datasets and having a pan-European reach, the ESID-R is likely to remain one of the most relevant scientific registries for patients with IEI.

## Materials and methods

### Technical background and operating mode of the ESID-R

The study protocol, the patient informed consent template and the center data transfer agreement plus amendments thereof were approved by the Institutional Review Board (IRB) and the data protection officer at the Medical University of Freiburg, Germany (Albert-Ludwigs-University). A substantial amendment was approved by the Medical University of Graz, Austria (24–334 ex 11/12, IRB00002556) and implemented in 2024. Details of the operational structure and technical background are found in the supplemental material.

### Data and statistical analyses

The study population included all patients with IEI/PID recorded in the ESID-R on March 19, 2024. Patients considered as discharged (*n* = 538), patients with secondary immunodeficiency who were documented as part of one national subregistry only (*n* = 417), or without definitive IEI diagnosis (*n* = 306) were excluded from the study, leaving 30,628 patients. IEI were classified using the latest classification of the IUIS (6), with abbreviations of the category names using, e.g., “PIRD,” typically referred to as primary immune regulatory diseases, as synonym for “Diseases of immune dysregulation” (category IV; i.e., including FHL). The statistical analyses were conducted using R (version 4.3.2). Continuous and categorical variables were reported using the medians and interquartile ranges, raw effectives, and percentages, respectively. Survival probabilities were estimated by applying the cumulative incidence function. Overall survival was defined as the time between birth and death from any cause. Curative therapies, such as allogeneic HSCT and GT, were considered as competing events. Survival was analyzed using the R package survival version 3.8–3 as described previously (32). As only patients with an eligible diagnosis are included, they are not considered to be at risk of dying before they are diagnosed (32). The prevalence was calculated based on the European population in 2019 (R package rnaturalearth version 1.0.1).

## Supplementary Material

Supplementary Material

Suppl. Figure 1A

Suppl. Figure 1B

Suppl. Figure 5

Suppl. Figure 1D

Suppl. Figure 1C

Suppl. Figure 3

Suppl. Figure 2

Suppl. Figure 4

Suppl. Figure 6

Suppl. Figure 7

Suppl. Table 1

Suppl. Table 2

Suppl. Table 3

Suppl. Table 4

Suppl. Table 5

Appendix_Study group_Collaborator List

Online supplemental material

In the supplement of this manuscript additional content is available relating to the evolution of the ESID-R and its content as well as supplementary figures as referenced in the text. Fig. S1 shows the history of patient inclusion into the ESID registry. Fig. S2 shows the visualization of ESID-R patient inclusion per population per country (minimal IEI/PID prevalence map). Fig. S3 shows the living status of patients at the age of last news in the ESID-R. Fig. S4 shows the IEI/PID distribution in an interactive, nested pie chart, showing details such as patient numbers with specific IEI/PID diagnoses at mouse-over and click to zoom. Fig. S5 shows the top 50 genetic diagnoses of IEI/PID patients in the ESID-R. Fig. S6 shows survival estimates separately for each IUIS category of IEI with confidence intervals. Fig. S7 shows countries in and around Europe with national patient registries used in addition to the ESID-R. Table S1 shows the contributing countries, sorted by the calculated prevalence per 100,000 inhabitants. Table S2 shows the underlying and main causes of death. Table S3 shows the therapy. Table S4 shows the sub-analysis of IEI/PID diagnoses in patients who died before the age of 5 years in two subcategories of IEI/PID. Table S5 shows the institutions of participants of the 2024 ESID-R survey on the international IEI/PID registry landscape.

## Figures and Tables

**Table 1.** ESID-R patient characteristics

Covariate	Values	Whole cohort	CID (I)	Syndromic (II)	PAD (III)	PIRD (IV)	Phagocyte (V)	Innate (VI)	AIS (VII)	Complement (VIII)	BMF (IX)	Phenocopies (X)	other
		*N* = 30,628	*N* = 2,531	*N* = 4,239	*N* = 15,123	*N* = 2,171	*N* = 2,548	*N* = 823	*N* = 1,042	*N* = 1,482	*N* = 85	*N* = 30	*N* = 554

Gender	M	16,767 (54.8%)	1,470 (58.1%)	2,473 (58.3%)	7,782 (51.5%)	1,329 (61.2%)	1,693 (66.5%)	387 (47%)	575 (55.2%)	681 (46%)	57 (67.1%)	19 (63.3%)	301 (54.3%)
	F	13,855 (45.2%)	1,061 (41.9%)	1,766 (41.7%)	7,337 (48.5%)	841 (38.8%)	854 (33.5%)	436 (53%)	467 (44.8%)	801 (54%)	28 (32.9%)	11 (36.7%)	253 (45.7%)

Year of birth		1997 (1976; 2008)	2007 (1997; 2014)	2005 (1995; 2012)	1984 (1963; 2001)	2004 (1993; 2001)	2002 (1991; 2010)	2003 (1991; 2011)	2008 (1999; 2013)	1991 (1972; 2006)	2004 (1987; 2011)	2001.5 (1995; 2008.5)	1998 (1974; 2007)

Familial case	N	21,854 (76.6%)	1,725 (72.9%)	3,002 (74.1%)	11,949 (85.4%)	1,266 (61.1%)	1,468 (65.4%)	492 (62.2%)	771 (76.6%)	645 (46.5%)	57 (75%)	28 (93.3%)	451 (87.7%)
	Y	6,681 (23.4%)	641 (27.1%)	1,047 (25.9%)	2,049 (14.6%)	805 (38.9%)	778 (34.6%)	299 (37.8%)	236 (23.4%)	742 (53.5%)	19 (25%)	2 (6.7%)	63 (12.3%)

Consanguinity	N	24,731 (87.2%)	1,426 (60.8%)	3,247 (82.1%)	13,381 (95.5%)	1,602 (78.8%)	1,699 (75.6%)	589 (77.3%)	920 (93.2%)	1,303 (93.1%)	66 (84.6%)	28 (100%)	470 (92.7%)
	Y	3,624 (12.8%)	921 (39.2%)	708 (17.9%)	631 (4.5%)	432 (21.2%)	547 (24.4%)	173 (22.7%)	67 (6.8%)	96 (6.9%)	12 (15.4%)	0 (0%)	37 (7.3%)

Age at onset of symptoms		3 (0.4; 14.1)	0.3 (0; 1.6)	0.5 (0; 2.2)	8 (2; 30)	2.2 (0.3; 7.5)	0.7 (0.1; 3)	1.2 (0.2; 3.8)	2.8 (0.7; 5)	8 (3; 18)	3 (0.3; 8.1)	2.8 (0.6; 4.9)	3 (0.7; 15.4)

Immune dysregulation at onset	N	21,482 (77.2%)	1,712 (77.5%)	3,242 (84.1%)	11,175 (80.3%)	616 (31.6%)	1,944 (86.2%)	632 (84.9%)	536 (55%)	1,182 (88.3%)	49 (67.1%)	4 (13.3%)	390 (80.7%)
	Y	6,347 (22.8%)	498 (22.5%)	613 (15.9%)	2,741 (19.7%)	1,335 (68.4%)	311 (13.8%)	112 (15.1%)	438 (45%)	156 (11.7%)	24 (32.9%)	26 (86.7%)	93 (19.3%)

Infection at onset	N	8,133 (28.9%)	436 (19.6%)	2,195 (56.6%)	1,920 (13.6%)	1,161 (59.6%)	590 (26%)	108 (14.4%)	705 (72.5%)	827 (61.5%)	44 (60.3%)	19 (67.9%)	128 (26.3%)
	Y	19,961 (71.1%)	1,786 (80.4%)	1,680 (43.4%)	12,202 (86.4%)	787 (40.4%)	1,681 (74%)	643 (85.6%)	267 (27.5%)	518 (38.5%)	29 (39.7%)	9 (32.1%)	359 (73.7%)

Malignancy at onset	N	27,294 (98.3%)	2,180 (98.6%)	3,766 (97.8%)	13,620 (98.1%)	1,885 (97.1%)	2,235 (99.4%)	741 (99.7%)	969 (99.8%)	1,333 (99.8%)	69 (94.5%)	25 (89.3%)	471 (97.7%)
	Y	479 (1.7%)	30 (1.4%)	85 (2.2%)	269 (1.9%)	57 (2.9%)	13 (0.6%)	2 (0.3%)	2 (0.2%)	3 (0.2%)	4 (5.5%)	3 (10.7%)	11 (2.3%)

Syndromic manifestations at onset	N	24,549 (88.3%)	2,046 (92.6%)	1,651 (42.7%)	13,574 (97.7%)	1,798 (92.5%)	2,075 (92.3%)	683 (91.8%)	919 (94.6%)	1,274 (95.4%)	40 (54.8%)	28 (100%)	461 (95.4%)
	Y	3,253 (11.7%)	164 (7.4%)	2,220 (57.3%)	320 (2.3%)	145 (7.5%)	174 (7.7%)	61 (8.2%)	52 (5.4%)	62 (4.6%)	33 (45.2%)	0 (0%)	22 (4.6%)

Other onset	N	24,466 (87.9%)	1,992 (90.1%)	3,298 (85.5%)	12,947 (93.1%)	1,782 (91.6%)	2,086 (92.7%)	685 (92.1%)	545 (55.6%)	647 (48.1%)	59 (80.8%)	28 (96.6%)	397 (81.9%)
	Y	3,366 (12.1%)	220 (9.9%)	559 (14.5%)	963 (6.9%)	163 (8.4%)	164 (7.3%)	59 (7.9%)	436 (44.4%)	699 (51.9%)	14 (19.2%)	1 (3.4%)	88 (18.1%)

No clinical symptoms	N	27,605 (94.1%)	2,188 (91%)	3,770 (92.5%)	13,875 (95.9%)	1,926 (91.5%)	2,238 (92.3%)	735 (91.9%)	966 (97.2%)	1,326 (91.2%)	71 (91%)	28 (100%)	482 (97.6%)
	Y	1,725 (5.9%)	217 (9%)	306 (7.5%)	595 (4.1%)	180 (8.5%)	187 (7.7%)	65 (8.1%)	28 (2.8%)	128 (8.8%)	7 (9%)	0 (0%)	12 (2.4%)

Age at clinical diagnosis (CD) (years)		8.2 (2; 30.1)	0.7 (0.3; 4.6)	2.4 (0.4; 6.9)	22 (6.2; 44)	5.1 (1; 13)	2 (0.5; 7)	4.2 (1.1; 14)	5 (2.7; 11.4)	15 (5.2; 28.8)	5 (1.3; 13.7)	5.2 (3; 9.8)	7.5 (2.3; 34.1)

Delay between onset and CD (years)		1.3 (0.1; 5.1)	0.2 (0; 1.3)	0.8 (0; 3.9)	2.7 (0.7; 7.5)	0.4 (0; 3.4)	0.5 (0.1; 2.2)	1 (0.1; 5.6)	1.5 (0.5; 4)	1.1 (0.1; 7)	0.5 (0.1; 2.1)	0.9 (0.3; 3.9)	1.7 (0.3; 4.7)

Genetics	Mutation found	12,774 (44.5%)	1,470 (63.1%)	3,647 (88.8%)	2,251 (16%)	1,671 (80.8%)	1,693 (71.8%)	529 (68.1%)	662 (65.3%)	772 (55.6%)	49 (71%)	30 (100%)	0 (0%)
	No mutation found	2,852 (9.9%)	319 (13.7%)	79 (1.9%)	1,850 (13.1%)	195 (9.4%)	106 (4.5%)	81 (10.4%)	71 (7%)	27 (1.9%)	9 (13%)	0 (0%)	115 (24.7%)
	Not tested	11,956 (41.7%)	403 (17.3%)	325 (7.9%)	9,398 (66.8%)	125 (6%)	478 (20.3%)	97 (12.5%)	250 (24.7%)	569 (41%)	8 (11.6%)	0 (0%)	303 (65%)
	Pending	1,097 (3.8%)	136 (5.8%)	57 (1.4%)	576 (4.1%)	76 (3.7%)	80 (3.4%)	70 (9%)	31 (3.1%)	20 (1.4%)	3 (4.3%)	0 (0%)	48 (10.3%)

Age at genetic diagnosis (GD)		5.5 (1.1; 15)	0.9 (0.3; 6.7)	3.2 (0.4; 9.7)	9.4 (2.9; 25)	8.3 (1.8; 17.3)	4.4 (1.1; 12.7)	10 (3.2; 20.4)	8.4 (4; 22)	13.2 (5; 26.9)	8.2 (3.6; 17.4)	9.8 (5; 14.1)	NA (NA; NA)

Delay between CD and GD		0.3 (0; 2.9)	0.2 (0; 1.2)	0.2 (0; 1.7)	1.3 (0.1; 8.8)	0.2 (0; 2.2)	0.3 (0; 2.2)	0.8 (0.1; 5.9)	0.1 (0; 1.1)	0.2 (0; 1.6)	0.4 (0.1; 2.6)	1 (0.2; 4.9)	NA (NA; NA)

Reason of GD	Clinical	8,787 (88%)	975 (87.7%)	2,601 (90.8%)	1,629 (89.2%)	1,192 (82.2%)	1,081 (90.6%)	380 (85.4%)	515 (88.6%)	350 (77.8%)	39 (92.9%)	25 (96.2%)	0 (0%)
	FamilY	975 (9.8%)	77 (6.9%)	153 (5.3%)	170 (9.3%)	245 (16.9%)	100 (8.4%)	63 (14.2%)	65 (11.2%)	99 (22%)	3 (7.1%)	0 (0%)	0 (0%)
	Neonatal	114 (1.1%)	41 (3.7%)	45 (1.6%)	20 (1.1%)	2 (0.1%)	3 (0.3%)	0 (0%)	1 (0.2%)	1 (0.2%)	0 (0%)	1 (3.8%)	0 (0%)
	Prenatal	112 (1.1%)	19 (1.7%)	64 (2.2%)	7 (0.4%)	11 (0.8%)	9 (0.8%)	2 (0.4%)	0 (0%)	0 (0%)	0 (0%)	0 (0%)	0 (0%)

Sequencing method of GD	Gene sequencing	7,693 (83.3%)	848 (80.1%)	2,122 (82%)	1,421 (83.5%)	1,154 (85.3%)	970 (87.7%)	323 (77.5%)	459 (85.5%)	347 (84.6%)	26 (70.3%)	23 (95.8%)	0 (0%)
	Nongenetic definitive test	441 (4.8%)	35 (3.3%)	284 (11%)	11 (0.6%)	12 (0.9%)	59 (5.3%)	3 (0.7%)	0 (0%)	36 (8.8%)	1 (2.7%)	0 (0%)	0 (0%)
	Whole exome/genome sequencing	1,100 (11.9%)	176 (16.6%)	183 (7.1%)	270 (15.9%)	187 (13.8%)	77 (7%)	91 (21.8%)	78 (14.5%)	27 (6.6%)	10 (27%)	1 (4.2%)	0 (0%)

Malignancy at any time	N	18,176 (91.1%)	1,578 (93.4%)	2,491 (88.9%)	8,759 (89.4%)	1,595 (91.4%)	1,609 (95.6%)	569 (94.7%)	604 (98.1%)	632 (97.7%)	66 (90.4%)	16 (72.7%)	257 (89.9%)
	Y	1,783 (8.9%)	112 (6.6%)	312 (11.1%)	1,034 (10.6%)	150 (8.6%)	74 (4.4%)	32 (5.3%)	12 (1.9%)	15 (2.3%)	7 (9.6%)	6 (27.3%)	29 (10.1%)

Covid-19	N	5,380 (70.3%)	456 (81%)	801 (76.1%)	2,613 (64.1%)	434 (74.2%)	363 (81%)	130 (77.4%)	260 (74.9%)	177 (76.3%)	15 (78.9%)	6 (75%)	125 (79.6%)
	Y	2,273 (29.7%)	107 (19%)	251 (23.9%)	1,461 (35.9%)	151 (25.8%)	85 (19%)	38 (22.6%)	87 (25.1%)	55 (23.7%)	4 (21.1%)	2 (25%)	32 (20.4%)

Living status	Alive	20,680 (67.5%)	1,419 (56.1%)	2,580 (60.9%)	10,740 (71%)	1,519 (70%)	1,534 (60.2%)	546 (66.3%)	729 (70%)	1,127 (76%)	52 (61.2%)	22 (73.3%)	412 (74.4%)
	Deceased	3,216 (10.5%)	615 (24.3%)	676 (15.9%)	1,117 (7.4%)	356 (16.4%)	269 (10.6%)	87 (10.6%)	23 (2.2%)	18 (1.2%)	22 (25.9%)	3 (10%)	30 (5.4%)
	Lost to follow-up	6,732 (22%)	497 (19.6%)	983 (23.2%)	3,266 (21.6%)	296 (13.6%)	745 (29.2%)	190 (23.1%)	290 (27.8%)	337 (22.7%)	11 (12.9%)	5 (16.7%)	112 (20.2%)

Age at last follow-up/death		20.2 (9.4; 42.6)	8.7 (2; 18.7)	12.5 (6.3; 19.9)	35.4 (16.9; 55.9)	14.8 (6.5; 24.2)	13.7 (5.8; 23.6)	15 (7; 27.3)	11.2 (6; 20.9)	27.3 (13.6; 46.4)	14.6 (6.3; 23.7)	18.2 (12; 22.8)	19 (8.4; 44.1)

Living status (stops at CT)	Alive at last follow-up	24,222 (81.1%)	928 (37.1%)	3,144 (76.5%)	13,522 (92%)	1,313 (61%)	1,680 (67%)	695 (86.1%)	956 (95.1%)	1,463 (99.1%)	41 (49.4%)	25 (83.3%)	455 (90.6%)
	Deceased	2,226 (7.5%)	258 (10.3%)	496 (12.1%)	966 (6.6%)	186 (8.6%)	181 (7.2%)	74 (9.2%)	17 (1.7%)	11 (0.7%)	9 (10.8%)	3 (10%)	25 (5%)
	Curative therapy	3,431 (11.5%)	1,315 (52.6%)	470 (11.4%)	216 (1.5%)	653 (30.3%)	648 (25.8%)	38 (4.7%)	32 (3.2%)	2 (0.1%)	33 (39.8%)	2 (6.7%)	22 (4.4%)

Age at last follow-up (stops at CT)		19.4 (8; 42.6)	1.3 (0.5; 11)	11.4 (5.2; 19.3)	35.5 (16.8; 55.9)	12.1 (3.2; 22.7)	11.6 (4; 21.8)	14.6 (6.6; 27)	11.1 (5.9; 20.7)	27.4 (13.6; 46.5)	10 (5; 20.5)	18.2 (11; 22.8)	19 (7.8; 44.1)

Follow-up duration (years)		7.2 (2.6; 14)	4.6 (0.8; 12)	8 (2.9; 14)	7.9 (3.4; 14.5)	5.6 (1.7; 11.6)	7.7 (2; 17.2)	6.6 (1.7; 13.1)	4 (1.6; 7.9)	7.7 (2.1; 16.9)	6.7 (3.4; 10.6)	11.2 (5.2; 17.1)	5.2 (1.5; 11)

Quantitative covariates: median (Q1; Q3); qualitative covariates: effective (percentage); the order of subgroups from left to right follows the IEI categories I–X of the 2022 IUIS classification available on the end date chosen for inclusion; values Y for “yes/present” for real positive values, N for “no” is shown to reflect the size of the correct comparison group, excluding “unknown” replies; percentages represent the fraction of patients with available information, not of the entire cohort; ages and diagnostic delay periods in years; diagnosis via NBS is recorded as subcategory of “no clinical symptoms/lab abnormalities only,” where not all choices of the submenu are shown; examples of “nongenetic definitive tests” are FISH, MLPA, or CGH arrays. AIS, autoinflammatory syndromes; MLPA, multiplex ligand-dependent probe amplification; CGH, comparative genomic hybridization; CT, curative/definitive therapy.

## Data Availability

Patient-level data are not publicly available due to privacy rights. Data underlying the figures in this manuscript may be available upon reasonable request from the corresponding author.
