# Supplementary material for: Inborn errors of immunity: Manifestation, treatment, and outcome—an ESID registry 1994–2024 report on 30,628 patients
Source: J Hum Immun. 2025 Jul 17;1(3):e20250007. doi: 10.70962/jhi.20250007 (PMC12674179; doi:10.70962/jhi.20250007)
Supplement: Table S1 — shows the contributing countries, sorted by the calculated prevalence per 100,000 inhabitants. [file jhi_20250007_tables1.docx]

**Supplementary Table 1. Contributing countries, sorted by the calculated prevalence per 100,000 inhabitants.**

| **Country** | **Registered patients** | **Genetically diagnosed (%)** | **Registered patients/100,000 inhabitants** | **Prevalence /100,000 inhabitants** |
| --- | --- | --- | --- | --- |
| Slovakia | 794 | 38.41% | 14.56 | 14.32 |
| France | 9,113 | 48.6% | 13.59 | 10.99 |
| Belgium | 1,333 | 17.78% | 11.61 | 11.36 |
| Switzerland | 658 | 44.68% | 7.67 | 7.55 |
| Slovenia | 129 | 62.79% | 6.18 | 6.18 |
| Germany | 5,056 | 44.96% | 6.08 | 5.73 |
| United Kingdom | 3,949 | 31.22% | 5.91 | 5.51 |
| Ireland | 280 | 39.29% | 5.67 | 5.48 |
| Portugal | 569 | 47.1% | 5.54 | 5.00 |
| Czechia | 573 | 52.88% | 5.37 | 4.98 |
| Netherlands | 715 | 26.85% | 4.13 | 3.96 |
| Greece | 387 | 48.32% | 3.61 | 3.46 |
| Hungary | 315 | 47.94% | 3.22 | 2.68 |
| Austria | 286 | 51.05% | 3.22 | 3.03 |
| Bulgaria | 175 | 38.29% | 2.51 | 2.42 |
| Belarus | 228 | 82.02% | 2.41 | 1.92 |
| Lithuania | 63 | 6.35% | 2.26 | 2.12 |
| Spain | 1,008 | 50.2% | 2.14 | 2.01 |
| Poland | 813 | 66.42% | 2.14 | 1.86 |
| Estonia | 26 | 11.54% | 1.96 | 1.88 |
| Italy | 1,036 | 27.7% | 1.72 | 1.58 |
| Iran | 1,284 | 9.35% | 1.55 | 1.39 |
| Denmark | 77 | 25.97% | 1.32 | 1.31 |
| Serbia | 82 | 34.15% | 1.18 | 1.17 |
| Turkey | 874 | 39.02% | 1.05 | 0.98 |
| Croatia | 36 | 58.33% | 0.89 | 0.79 |
| Egypt | 512 | 43.95% | 0.51 | 0.41 |
| Ukraine | 140 | 82.86% | 0.32 | 0.29 |
| Romania | 50 | 76% | 0.26 | 0.20 |
| Israel | 5 | 80% | 0.06 | 0.06 |
| Russia | 59 | 93.22% | 0.04 | 0.03 |
| Bosnia and Herzegowina | 1 | 100% | 0.03 | 0.03 |
| Sweden | 2 | 100% | 0.02 | 0.02 |

Of note, the ESID-R has no means to influence the balance of data entry per country or center, resulting in differences of the proportion of children, adolescent and adult patients, the “weight” given to certain diseases at some centers and their potential overrepresentation. Although measures were taken and found to be successful to unify the classification of non-genetic diseases such as common variable immune deficiency or selective IgA deficiency by applying the ESID registry working definitions for making a clinical diagnosis, the variance in the “epidemiological activity” of some centers as compared to others remains.
