# Supplementary material for: Inborn errors of immunity: Manifestation, treatment, and outcome—an ESID registry 1994–2024 report on 30,628 patients
Source: J Hum Immun. 2025 Jul 17;1(3):e20250007. doi: 10.70962/jhi.20250007 (PMC12674179; doi:10.70962/jhi.20250007)
Supplement: Table S2 — shows the underlying and main causes of death. [file jhi_20250007_tables2.docx]

**Supplementary Table 2. Underlying and main causes of death.**

| **Covariate** | **Whole cohort** | **CID (I)** | **Syndromic (II)** | **PAD (III)** | **PIRD (IV)** | **Phagocyte (V)** | **Innate (VI)** | **AIS (VII)** | **Complement (VIII)** | **BMF (IX)** | **Phenocopies (X)** | **other** |
| --- | --- | --- | --- | --- | --- | --- | --- | --- | --- | --- | --- | --- |
|  | **N=30628** | **N=2531** | **N=4239** | **N=15123** | **N=2171** | **N=2548** | **N=823** | **N=1042** | **N=1482** | **N=85** | **N=30** | **N=554** |
| Underlying cause of death: Infection | 989 (32.8%) | 302 (51.3%) | 192 (31.7%) | 232 (22.2%) | 99 (29.3%) | 89 (34.6%) | 47 (54%) | 10 (43.5%) | 4 (28.6%) | 9 (40.9%) | 0 (0%) | 5 (17.9%) |
| Underlying cause of death: Malignancy | 456 (15.1%) | 34 (5.8%) | 154 (25.5%) | 199 (19%) | 28 (8.3%) | 22 (8.6%) | 8 (9.2%) | 0 (0%) | 3 (21.4%) | 1 (4.5%) | 3 (100%) | 4 (14.3%) |
| Underlying cause of death: Immune dysregulation | 350 (11.6%) | 47 (8%) | 54 (8.9%) | 90 (8.6%) | 122 (36.1%) | 16 (6.2%) | 7 (8%) | 7 (30.4%) | 2 (14.3%) | 1 (4.5%) | 0 (0%) | 4 (14.3%) |
| Underlying cause of death: transplantation | 371 (12.3%) | 154 (26.1%) | 48 (7.9%) | 30 (2.9%) | 87 (25.7%) | 33 (12.8%) | 9 (10.3%) | 4 (17.4%) | 2 (14.3%) | 3 (13.6%) | 0 (0%) | 1 (3.6%) |
| Underlying cause of death: Other | 434 (14.8%) | 46 (8.6%) | 104 (17.3%) | 197 (18.9%) | 22 (6.9%) | 30 (12.1%) | 22 (25.6%) | 3 (13%) | 1 (7.1%) | 5 (22.7%) | 0 (0%) | 4 (14.8%) |
| Main cause of death: Septic Shock | 405 (13.4%) | 82 (13.9%) | 75 (12.4%) | 127 (12.1%) | 37 (10.9%) | 38 (14.8%) | 33 (37.9%) | 8 (34.8%) | 3 (21.4%) | 2 (9.1%) | 0 (0%) | 0 (0%) |
| Main cause of death: Renal Failure | 54 (1.8%) | 10 (1.7%) | 12 (2%) | 20 (1.9%) | 6 (1.8%) | 3 (1.2%) | 2 (2.3%) | 0 (0%) | 0 (0%) | 1 (4.5%) | 0 (0%) | 0 (0%) |
| Main cause of death: Neurological Complications | 183 (6.1%) | 28 (4.8%) | 24 (4%) | 48 (4.6%) | 43 (12.7%) | 18 (7%) | 15 (17.2%) | 2 (8.7%) | 1 (7.1%) | 1 (4.5%) | 0 (0%) | 3 (10.7%) |
| Main cause of death: Relapse of Malignancy | 200 (6.6%) | 17 (2.9%) | 55 (9.1%) | 89 (8.5%) | 16 (4.7%) | 12 (4.7%) | 5 (5.7%) | 0 (0%) | 1 (7.1%) | 2 (9.1%) | 2 (66.7%) | 1 (3.6%) |
| Main cause of death: GvHD | 82 (2.7%) | 44 (7.5%) | 10 (1.7%) | 5 (0.5%) | 12 (3.6%) | 10 (3.9%) | 1 (1.1%) | 0 (0%) | 0 (0%) | 0 (0%) | 0 (0%) | 0 (0%) |
| Main cause of death: PTLD | 25 (0.8%) | 10 (1.7%) | 7 (1.2%) | 1 (0.1%) | 5 (1.5%) | 1 (0.4%) | 1 (1.1%) | 0 (0%) | 0 (0%) | 0 (0%) | 0 (0%) | 0 (0%) |
| Main cause of death: Heart Failure | 125 (4.2%) | 17 (2.9%) | 23 (3.8%) | 49 (4.7%) | 13 (3.8%) | 14 (5.4%) | 3 (3.4%) | 3 (13%) | 1 (7.1%) | 0 (0%) | 2 (66.7%) | 0 (0%) |
| Main cause of death: Multiple Organ Failure | 368 (12.2%) | 89 (15.1%) | 64 (10.6%) | 83 (7.9%) | 74 (21.9%) | 31 (12.1%) | 19 (21.8%) | 3 (13%) | 0 (0%) | 4 (18.2%) | 0 (0%) | 1 (3.6%) |
| Main cause of death: Surgical Complications | 19 (0.6%) | 1 (0.2%) | 7 (1.2%) | 5 (0.5%) | 0 (0%) | 3 (1.2%) | 2 (2.3%) | 1 (4.3%) | 0 (0%) | 0 (0%) | 0 (0%) | 0 (0%) |
| Main cause of death: Veno Occlusive Disorder | 22 (0.7%) | 11 (1.9%) | 2 (0.3%) | 0 (0%) | 6 (1.8%) | 2 (0.8%) | 0 (0%) | 1 (4.3%) | 0 (0%) | 0 (0%) | 0 (0%) | 0 (0%) |
| Main cause of death: Graft Rejection | 45 (1.5%) | 21 (3.6%) | 5 (0.8%) | 3 (0.3%) | 7 (2.1%) | 5 (1.9%) | 2 (2.3%) | 1 (4.3%) | 0 (0%) | 0 (0%) | 0 (0%) | 1 (3.6%) |
| Main cause of death: Respiratory Failure | 640 (21.2%) | 156 (26.5%) | 174 (28.8%) | 160 (15.3%) | 70 (20.7%) | 49 (19.1%) | 10 (11.5%) | 6 (26.1%) | 3 (21.4%) | 6 (27.3%) | 3 (100%) | 3 (10.7%) |
| Main cause of death: Haemorrhage | 119 (4%) | 15 (2.5%) | 30 (5%) | 36 (3.4%) | 20 (5.9%) | 7 (2.7%) | 6 (6.9%) | 3 (13%) | 0 (0%) | 1 (4.5%) | 0 (0%) | 1 (3.6%) |
| Main cause of death: Drug Toxicity | 11 (0.4%) | 2 (0.3%) | 3 (0.5%) | 4 (0.4%) | 2 (0.6%) | 0 (0%) | 0 (0%) | 0 (0%) | 0 (0%) | 0 (0%) | 0 (0%) | 0 (0%) |
| Main cause of death: Liver Failure | 72 (2.4%) | 10 (1.7%) | 14 (2.3%) | 34 (3.3%) | 8 (2.4%) | 3 (1.2%) | 3 (3.4%) | 0 (0%) | 0 (0%) | 0 (0%) | 0 (0%) | 0 (0%) |
| Main cause of death: Thrombosis | 5 (0.2%) | 1 (0.2%) | 0 (0%) | 4 (0.4%) | 0 (0%) | 0 (0%) | 0 (0%) | 0 (0%) | 0 (0%) | 0 (0%) | 0 (0%) | 0 (0%) |
| Main cause of death: Other | 238 (7.9%) | 24 (4.1%) | 35 (5.8%) | 103 (9.8%) | 41 (12.1%) | 20 (7.8%) | 8 (9.2%) | 2 (8.7%) | 3 (21.4%) | 0 (0%) | 0 (0%) | 2 (7.1%) |

Effective (Percentage); the order of subgroups from left to right follows the IEI categories I-X of the 2022 IUIS classification available on the end date chosen for inclusion with abbreviations for combined immunodeficiencies (CID), CID with syndromic features (Syndromic), primary antibody deficiencies (PAD), diseases with immune dysregulation or primary immune regulatory disorders (PIRD), diseases of phagocyte number or function (Phagocyte), disorders of innate immunity (Innate), autoinflammatory syndromes (AIS), complement deficiencies (Complement), bone marrow failure syndromes (BMF), and phenocopies.
