# Supplementary material for: Inborn errors of immunity: Manifestation, treatment, and outcome—an ESID registry 1994–2024 report on 30,628 patients
Source: J Hum Immun. 2025 Jul 17;1(3):e20250007. doi: 10.70962/jhi.20250007 (PMC12674179; doi:10.70962/jhi.20250007)
Supplement: Table S3 — shows the therapy. [file jhi_20250007_tables3.docx]

**Supplementary Table 3. Therapy.**

| **Covariate** | **Values** | **Whole cohort** | **CID (I)** | **Syndromic (II)** | **PAD (III)** | **PIRD (IV)** | **Phagocyte (V)** | **Innate (VI)** | **AIS (VII)** | **Complement (VIII)** | **BMF (IX)** | **Phenocopies (X)** | **other** |
| --- | --- | --- | --- | --- | --- | --- | --- | --- | --- | --- | --- | --- | --- |
|  |  | **N=30628** | **N=2531** | **N=4239** | **N=15123** | **N=2171** | **N=2548** | **N=823** | **N=1042** | **N=1482** | **N=85** | **N=30** | **N=554** |
| Currently under IgRT | N | 13701 (57.5%) | 1039 (63.8%) | 2280 (74.9%) | 4316 (34.9%) | 1290 (79.2%) | 1864 (96.5%) | 525 (82.9%) | 770 (93.4%) | 1269 (98.2%) | 48 (85.7%) | 20 (95.2%) | 280 (73.9%) |
|  | Y | 10107 (42.5%) | 589 (36.2%) | 764 (25.1%) | 8054 (65.1%) | 339 (20.8%) | 68 (3.5%) | 108 (17.1%) | 54 (6.6%) | 23 (1.8%) | 8 (14.3%) | 1 (4.8%) | 99 (26.1%) |
| IgRT: route | Intravenous | 6410 (51%) | 572 (65.3%) | 600 (58.8%) | 4643 (48.2%) | 324 (63.9%) | 87 (73.1%) | 66 (43.7%) | 41 (57.7%) | 11 (33.3%) | 8 (66.7%) | 2 (66.7%) | 56 (41.2%) |
|  | Subcutaneous | 6119 (48.7%) | 297 (33.9%) | 416 (40.8%) | 4973 (51.6%) | 181 (35.7%) | 32 (26.9%) | 85 (56.3%) | 30 (42.3%) | 21 (63.6%) | 4 (33.3%) | 1 (33.3%) | 79 (58.1%) |
|  | Intramuscular | 17 (0.1%) | 1 (0.1%) | 2 (0.2%) | 12 (0.1%) | 1 (0.2%) | 0 (0%) | 0 (0%) | 0 (0%) | 1 (3%) | 0 (0%) | 0 (0%) | 0 (0%) |
|  | Push (s.c.) | 24 (0.2%) | 6 (0.7%) | 2 (0.2%) | 14 (0.1%) | 1 (0.2%) | 0 (0%) | 0 (0%) | 0 (0%) | 0 (0%) | 0 (0%) | 0 (0%) | 1 (0.7%) |
| Age at first IgRT |  | 19.3 [5.2;41.8] | 2 [0.5;10.3] | 5 [1.3;11.5] | 28.6 [8.7;46.7] | 10.2 [4.2;19.1] | 6.3 [2.1;11.8] | 6.7 [2.3;13.3] | 5.6 [1.9;15.9] | 9.7 [5.6;18] | 1.6 [0.7;7.3] | 7 [6.1;14] | 17.1 [5.9;45.2] |
| AlloSCT | N | 27263 (89%) | 1261 (49.8%) | 3783 (89.2%) | 14907 (98.6%) | 1518 (69.9%) | 1907 (74.8%) | 785 (95.4%) | 1010 (96.9%) | 1480 (99.9%) | 52 (61.2%) | 28 (93.3%) | 532 (96%) |
|  | Y | 3365 (11%) | 1270 (50.2%) | 456 (10.8%) | 216 (1.4%) | 653 (30.1%) | 641 (25.2%) | 38 (4.6%) | 32 (3.1%) | 2 (0.1%) | 33 (38.8%) | 2 (6.7%) | 22 (4%) |
| Age at first AlloSCT |  | 2 [0.6;8.1] | 0.7 [0.4;1.7] | 2.3 [1.1;6.5] | 9.5 [3.5;17.7] | 2.4 [0.5;8.9] | 6.1 [2.2;13.1] | 7.8 [3.3;10.7] | 4 [1.5;7] | 11.4 [10.5;12.3] | 10 [5.1;16] | 7.9 [6.6;9.2] | 7.4 [4.9;14.4] |
| Gene therapy | N | 29070 (99.7%) | 2327 (97.9%) | 4011 (99.6%) | 14465 (100%) | 2086 (100%) | 2452 (99.6%) | 793 (100%) | 923 (100%) | 1411 (100%) | 81 (100%) | 30 (100%) | 491 (100%) |
|  | Y | 77 (0.3%) | 50 (2.1%) | 17 (0.4%) | 0 (0%) | 0 (0%) | 10 (0.4%) | 0 (0%) | 0 (0%) | 0 (0%) | 0 (0%) | 0 (0%) | 0 (0%) |
| Age at first gene therapy |  | 2.3 [0.8;4.6] | 0.9 [0.6;2.9] | 3.6 [2.8;7.4] | NA [NA;NA] | NA [NA;NA] | 15.1 [7.7;22.9] | NA [NA;NA] | NA [NA;NA] | NA [NA;NA] | NA [NA;NA] | NA [NA;NA] | NA [NA;NA] |
| Splenectomy | N | 19732 (97.8%) | 1565 (98.1%) | 2681 (96.8%) | 9846 (97.9%) | 1620 (94.8%) | 1485 (99.3%) | 603 (99.7%) | 753 (99.1%) | 789 (99.9%) | 55 (100%) | 21 (84%) | 314 (98.7%) |
|  | Y | 453 (2.2%) | 30 (1.9%) | 90 (3.2%) | 216 (2.1%) | 89 (5.2%) | 10 (0.7%) | 2 (0.3%) | 7 (0.9%) | 1 (0.1%) | 0 (0%) | 4 (16%) | 4 (1.3%) |
| Age at splenectomy |  | 17.9 [7.3;33.3] | 16 [9;24] | 5.4 [2.3;8] | 31.3 [20.1;42] | 11.3 [6.7;20.2] | 8.9 [4.5;21.3] | 2.8 [2.8;2.8] | 21.2 [17.9;36.1] | 33.9 [33.9;33.9] | NA [NA;NA] | 7.2 [4.6;8.6] | 19.3 [15.7;25.3] |

Quantitative covariates: Median [Q1;Q3]; qualitative covariates: Effective (Percentage)
