# Supplementary material for: Inborn errors of immunity: Manifestation, treatment, and outcome—an ESID registry 1994–2024 report on 30,628 patients
Source: J Hum Immun. 2025 Jul 17;1(3):e20250007. doi: 10.70962/jhi.20250007 (PMC12674179; doi:10.70962/jhi.20250007)
Supplement: Table S4 — shows the sub-analysis of IEI/PID diagnoses in patients who died before the age of 5 years in two subcategories of IEI/PID. [file jhi_20250007_tables4.docx]

**Supplementary Table 4. Sub-analysis of IEI/PID diagnoses in patients who died** **before the age of 5 years in two subcategories of IEI/PID.**

| IEI/PID diagnoses underlying death before the age of 5 years in the category *diseases with immune dysregulation* (synonymously used as primary immune regulatory disorders, *PIRD*) \| *n*= | |
| --- | --- |
| Familial hemophagocytic lymphohistiocytosis syndromes (FHLH)* | 44 |
| X-linked lymphoproliferative syndrome (XLP)* | 10 |
| FOXP3 deficiency (IPEX) | 4 |
| Autoimmune lymphoproliferative syndrome (ALPS) | 3 |
| Early-onset inflammatory bowel disease | 2 |
| Griscelli syndrome, type 2* | 2 |
| Unclassified disorders of immune dysregulation | 2 |
| APECED / APS1 with CMC - Autoimmune polyendocrinopathy candidiasis ectodermal dystrophy (APECED) | 1 |
| Chediak Higashi syndrome* | 1 |
| IPEX-like disease | 1 |
| *proportion of patients with risk of hemophagocytic lymphohistiocytosis (HLH) | 81·4% |
|  | |
| IEI/PID diagnoses underlying death before the age of 5 years in category *disorders of innate or intrinsic immunity (*abbreviated *Innate)* \| *n*= | |
| Defects of TLR/NFkappa-B signalling | 7 |
| Isolated congenital asplenia | 7 |
| Defects with susceptibility to mycobacterial infection (MSMD) | 4 |
| ZNFX1 deficiency | 2 |
| Asplenia syndrome (Ivemark syndrome) | 1 |
| Chronic mucocutaneous candidiasis (CMC) | 1 |
| IRF9 deficiency | 1 |
| Unclassified defects in innate immunity | 1 |
