# Supplementary material for: Inborn errors of immunity: Manifestation, treatment, and outcome—an ESID registry 1994–2024 report on 30,628 patients
Source: J Hum Immun. 2025 Jul 17;1(3):e20250007. doi: 10.70962/jhi.20250007 (PMC12674179; doi:10.70962/jhi.20250007)
Supplement: Table S5 — shows the institutions of participants of the 2024 ESID-R survey on the international IEI/PID registry landscape. [file jhi_20250007_tables5.docx]

**Supplementary Table 5. Institutions of participants of the 2024 ESID-R survey on the international IEI/PID registry landscape.**

| **Western Europe** | **Eastern Europe** | **Other countries** |
| --- | --- | --- |
| Denmark (Copenhagen) | Armenia (Yerevan) | Australia (Melbourne) |
| Finland (Helsinki) | Belarus (Minsk) | Brazil (São Paulo) |
| Germany  (Dresden, Freiburg, Leipzig, Munich) | Bulgaria (Sofia) | Canada (Halifax) |
| Ireland (Dublin) | Czech Republic (Prague) | Egypt (Cairo) |
| Italy (Milan) | Georgia (Tbilisi) | Iran (Teheran) |
| Portugal (Coimbra) | Hungary (Budapest) | Sudan (Khartoum) |
| Spain (Barcelona) | Latvia (Riga) | Turkey (Istanbul) |
| Sweden (Jönköping) | Romania (Bucharest) | USA (Philadelphia) |
| Switzerland (Geneva, Zurich) | Russia (Moscow) |  |
| United Kingdom (Sheffield) | Serbia (Belgrade) |  |
|  | Ukraine (Kyiv) |  |

*The survey participants chose to reply (or not) to a survey which had been distributed via the ESID-R listserv and was also posted on the website during the summer of 2024.*
