# Supplementary material for: Inborn errors of immunity: Manifestation, treatment, and outcome—an ESID registry 1994–2024 report on 30,628 patients
Source: J Hum Immun. Author manuscript; Available in PMC 2025 Dec 4. (PMC12674179; doi:10.70962/jhi.20250007)
Supplement: Supplementary file 1 [file NIHMS2107117-supplement-Appendix_Study_group_Collaborator_List.pdf]

Appendix

ESID-R Working Party members: Dalia Abd Elaziz, Sohilla Lofty M. Abdelkader, Avigaelle Abitbol, Hassan Abolhassani, Lalash Abrahim, Mohamed Abuzakouk, Pietro Andrea Accardo, Nabila Achir Moussouni, Veronica Afonso, Philipp Agyeman, Martina Ahlmann, Alessandro Aiuti, Abla Akl, Güzide Aksu, Kim Albers, Meda Diana Alecsandru, Olga Aleinikova, Svetlana Aleshkevich, Radwa Salah Eldeen Youssif Alkady, Luis Allende, Zoe Allwood, Manrique de Lara Laia Alsina, Daniela Ambrosch-Barsoumian, Lisa Ameshofer, Kenza Amour, Evrim Anadol, Ariharan Ananthachagaran, Chantal Andriamanga, Julia Andris, Karin Andritschke, Federica Angelini, Tobias Ankermann, Katrin Apel, Siamak Arami, Ömür Ardeniz, Peter Arkwright, Karina Arnold, Rudolf Ascherl, Najla Assam, Nurit Assia-Batzir, Faranaz Atschekzei, Sybille Aumann, Volker Aumann, Magnus Aurivillius, Bernd Ausserer, Tadej Avcin, Sezin Aydemir, Emel Aygören-Pürsün, Cagdas Ayvaz Deniz, Chiara Azzari, Lo Babacar, Perrine Bach, Sophie Bachmann, Peter Bader, Shahrzad Bakhtiar, Renate Bancé, Catherine Bangs, Katerina Barfusz, Safa Baris, B. Barlan Isil, Michaela Bartsch, Lucia Augusta Baselli, Laura Batlle-Maso, Elena Bertolini, Helge Baumann, Ulrich Baumann, Veronika Baumeister, Helen Baxendale, Suzanne Bazen, Beatrice Beaurain, Julien Beauté, Mounia Bechar-Makhloufi, Norbert Beck, Brigitta Becker, Christian Becker, Uta Behrends, Renata Beider, Rita Beier, Amel Belkacem, Luisa Belke, Sven Bellert, Bernd H. Belohradsky, Lilia Ben Slama, Aouatef Ben-Bouzid, Vincent Benoît, Thamila Berdous-Sahed, Jan Bergils, Anna Berglöf, Peter Bergman, Katarzyna Bernat-Sitarz, Jolanta Bernatoniene, Ewa Bernatowska, Benedikt Bernbeck, Claire Bethune, Kai Beuckmann, Malini Bhole, Anika-Kerstin Biegner, Stefan Bielack, Kirsten Bienemann, Arndt Bigl, Amélie Bigorgne, Marc Bijl, Nadine Binder, Michaela Bitzenhofer-Grüber, Geraldine Blanchard Rohner, Dagmar Blank, Claudia Blattmann, Julia Blau, Audra Blaziene, Stefan Blazina, Markéta Bloomfield, Roswitha Blume, Barbara Boardman, Sebastian Bode, Jaap-Jan Boelens, Christoph Boesecke, Delfien Bogaert, Johannes Bogner, Nadezda Bohynikova, Claire Booth, Victoria Bordon, Arndt Borkhardt, Melanie Börries, Michael Borte, Stephan Borte, Lukas Bossaller, Madeleine Bossard, Jeannet Botros, Soraya Boucherit, Jeannette Boutros, Oksana Boyarchuk, Onur Boyman, Kaan Boztug, Sara Branco Pereira da Silva, Thomas Braschler, Sophie Bravo, Robbert Bredius, Philip Bright, Carolina Brito de Azevedo Amaral, Nicholas Brodszki, Grit Brodt, Allan Brolund, Pauline Brosselin, Bastian Brummel, Sonja Brun-Schmid, Jürgen Brunner, Roswitha Bruns, Christina Buchta, Dietke Buck, Aileen Bücker, Martina Bührlen, Stefan Burdach, Siobhan Burns, Janet Burton, Luis Caminal, Caterina Cancrini, Clementina Canessa, Nathan Cantoni, Brindusa Capilna, Fabiola Caracseghi, Isabel Caragol, Javier Carbone, Maria Carrabba, Latanya Chamberlain, Anita Chandra, Christophe Chantrain, Helen Chapel, Julie Chassot, Ronnie Chee, Matteo Chinello, Charu Chopra, Zita Chovancova, Martin Christmann, Krystyna Chrzanowska, Peter Ciznar, Karlien Claes, Carl Friedrich Classen, Martin Classen, Alexis-Virgil Cochino, Alison M. Condliffe, Selim Corbacioglu, Ana Isabel Cordeiro, Donatienne Cordier Wynar, Claire Core, Laurence Costes, Tanya Coulter, Virginie Courteille, Maria Cristina, Maria Cucuruz, Nel Dabrowska Leonik, Mandy Dähling, Mary Louise Daly, Claudia-Sabrina Daniel, Maria Giovanna Danieli, James Darroch, Graham Davies, Frans de Baets, Christiane De Boeck, Javier De Gracia Roldan, Narimene de Nadai, Iris de Schutter, Nathalie De Vergnes, Esther de Vries, Josine de Witte, Theo Debert, Corina Defila, Judith Deimel, Diane Delaplace, Rosa Maria Dellepiane, Nelli Dellert, Laura Delliera, Anita Delor, Ulrike Demel, John Dempster, M.M. den Os, Rosmarie Dengg, Sora Asfaw Desisa, Alexandra Desta, Drahomira Detkova, Maite Dewerchin, Romina Dieli Crimi, Dagmar Dilloo, Florentia Dimitriou, Sarah Svenja Dinges, Jasmin Dinser, Nabila Dipani, Johannes Dirks, Anna-Maria Dittrich, Lylia Djermane, Yagmur Dogru, Figen Dogu Esin, Angelika Dombrowski, Julia Dominguez Escobar, Michaela Döring, Camilla Heldbjerg Drabe, Susann Drerup, Barbara Drexel, G.J.A. Driessen, Gregor Dückers, Yasmine Dudoit, Andrea Duppenthaler, Georg Ebetsberger-Dachs, Mate Barnabas Ecser, Maartje Eekman, Rosanna Ehrat, Eva Eisl, Olov Ekwall, Rabab El Hawary, Sabine M. El-Helou, Aisha El-Marsafy, Sarina Elbe, Suzanne Elcombe, Alia Eldash, Youssif Alkady Radwa Salah Eldeen, P.M. Ellerbroek, Roland Elling, Jane Elliott, Angelika Engelhardt, Diana Ernst, Fügen Ersoy, Stefanie Esper, Isabel Esteves, Andrew Exley, Martin Faber, Giovanna Fabio, Gaby Fahrni, Laura Eva Faletti, Laura Eva Faletti, Claire-Michele Farber, João Farela Neves, Emilia Faria, Henriette Farkas, Evangelia Farmaki, Maria Faßhauer, Anders Fasth, Gerd Fätkenheuer, Stefanie Faustmann, Gisela Fecker, Conleth Feighery, Cornelia Feiterna-Sperling, Perez Eduardo Fernandez-Cruz, Cordeiro Ferreira Concalo, Alice Ferster, Tobias Feuchtinger, Oliver Feyen, Daniela Finke, Sigrid Fitter, Stefan Flaschberger, Lucia Fleckenstein, Dirk Föll, Adriano Fontana, Concetta Forino, Elisabeth Förster-Waldl, Nora Franchet, Dagmar Freitag, Urs P. Frey, Hannah Margarete Frick, Elisabeth Friedel, Wilhelm Friedrich, Barbara Frisch, Lukas Frischknecht, Stephanie Fritzemeyer, Alenka Gagro, Manfred Gahr, Nermeen Mouftah Galal, Eleonora Gambineri, Agnes Gamper, Franziska Gams, Astrid Ganzow, Nicolas Garcelon, Tomaz Garcez, Marina Garcia Prat, Ann Gardulf, Janine Garibay, Birgit Garwer, Jonathan Gathmann, Corinna Gebauer, Linda Geberzahn, Tilman Geikowski, Ulf Geisen, Christiane Gemander, Andrew R. Gennery, Marie Gerisch, Michael Gernert, Katrin Gerrer, Stev Gerschmann, Carolin Giannini, Juana Gil Herrera, Noemi Gimenez Sanz, Matthias Girndt, Ramona Girrbach, Hermann Girschick, Ioannis Gkougkourelas, Dominika Gładysz, Susanne Gnatowski, Elisabeth Gnodtke, Vera Goda, Sarah Goddard, Daniela Goebel, Jean-Christophe Goffard, Sigune Goldacker, Eva Maria Gollowitsch, Manuella Gomes, Mark Gompels, Míriam González, Luis Ignacio Gonzalez Granado, Pavels Gordins, Katharina Gößling, Lisa Göschl, Lucy Gossens, Ewelina Gowin, Lea Graafen, Leo Graca, Brigita Gradauskiene (Sitkauskiene), Dagmar Graf, Norbert Graf, Elliot Grange, H. Anne Grashoff, Johann Greil, Sofia Grigoriadou, Helen Gronlund, Ute Groß-Wieltsch, Teresa Guerra, Kissy Guevara-Hoyer, Mor Seny Gueye, Noemie Guibert, Birgit Gülnur, Tayfun Güngör, Marina Guseva, David Guzman, Marcel Haag, Gabriele Haase, Henriette Haenicke, Filomeen Haerynck, Ines Hafsa, David Hagin, Emine Haliti, Michael Hallek, Gonca Hancioglu, Rupert Handgretinger, Leif G. Hanitsch, Susanne Hansen, Tetyana Hariyan, Thomas Harrer,

Downloaded from http://rupress.org/jhi/article-pdf/1/3/e20250007/1947361/jhi_20250007.pdf by guest on 16 November 2025

https://doi.org/10.70962/jhi.20250007

Pia Hassunah, Maria Hatzistilianou, Fabian Hauck, Thomas Hauser, Margje H. Haverkamp, Grant Hayman, Paul Heath, Christian Hedrich, Maximilian Heeg, Michael Heike, Martin Heimbrodt, Sabine Heine, Ulrich Heininger, Christian Heinrich, Valerie Heinz, Andreas Heitger, Matthew Helbert, Arthur Helbling, Camilla Heldbjerg Drabe, Antje Hellige, Julya Hempel, Karen Henderson, Jörg Henes, Philipp Henneke, Christian Hennig, Karin Henrichs, Martin Herbst, Walter Hermann, Manuel Hernandez, Anja Hernández, Edyta Heropolitanska, Edyta Heropolitanska-Pliszka, Richard Herriot, Friedrich Herrmann, Archana Herwadkar, Christoph Hess, Ursula Hess, Sebastian Hesse, Sonja Higgins, Anna Hilfanova, Sophie Hilpert, Chantal Hintze, Eva Hlavackova, Eva Hlaváčková, Isabel Hodl, Adna Hodzic, Miriam Hoernes, Christina Hoffmann, Andreas Holbro, Christiane Höllinger, Lisa Holtsch, Ursula Holzer, Dirk Holzinger, Manfred Hönig, Andrea Hönscheid, Julia Horn, Gerd Horneff, Claire Hoyoux, Nataliya Hristova, Kai Hübel, Angela Hübner, Christian Huemer, Aarnoud Huissoon, Brigitte Hülsmann, Patrick Hundsdörfer, Hans-Iko Huppertz, Kristina Huß, Sadia Hussain, Julien Husson, Tanja Hüttner-Foehlisch, Hanna Ijspeert, Aydan Ikinciogullari, Kökçü İlknur, Ninela Irga, Alexandra Jablonka, Karina Jahnz-Rozyk, Marcus Jakob, Donate Jakoby-Gaide, Peter Jandus, Annette Jansson, Melanie Jaquet, Helene Jardefors, Edyta Jargulinska, Barbara Jauk, Milos Jesenak, Katharina Jilka, Stephen Jolles, Alison Jones, Regina Jones, Birgit Jonkman-Berk, Göran Jönsson, Hilary J. Joyce, Pricillia Juliana, Michael Kabesch, Leo Kager, Christian Kahlert, Petra Kaiser-Labusch, Ioannis Kakkas, Dirk Kamitz, Maria Kanariou, Lothar Kanz, Elif Karakoc-Aydiner, Boris Karanovic, Mutlu Kartal-Kaess, Elisabeth Käser, Terese L. Katzenstein, Hana Kayserova, Peter Kelleher, Tessa Kerre, Sara Sebnem Kilic, Martina Kirchner, Simone Kiwit, Ayca Kiykim, Jessica Klasen, Maja Klaudel-Dreszler, Ariane Klein, Christoph Klein, Andreas Klein-Franke, Ilona Kleine, Stefan Kleinert, Christian Klemann, Marion Klima, Adam Klocperk, Robin Kobbe, Dilara Fatma Kocacik Uygun, Melanie Koch, Yvonne Kochler, Naschla Kohistani, Marina Kojic, Antonio Kolios, Uwe Kölsch, Sylwia Koltan, Irina Kondratenko, Christoph Königs, Julia Konoplyannikova, Peter Kopac, Jana Kopp, Dieter Körholz, Julia Körholz, Pauline Korte, Larysa Kostyuchenko, Ina Kötter, Sven Kracker, Pavlina Králícková, Christof Kramm, Philipp Kramme, Máté Krausz, Wolfhart Kreuz, Johanna Krista, Gergely Kriván, Gabriele Kropshofer, Renate Krüger, Olga Krystufkova, Maria Ktistaki, Jörn-Sven Kühl, Alexander Kühn, Taco W. Kuijpers, Wietse Kuis, Silke Kullmann, Andreas Kulozik, Dinakantha Kumararatne, Ria Kümmler, Andrea Kündgen, Magdalena Kurenko – Deptuch, Cosima Kuss Paula, Necil Kütükcüler, Cécile Lafoix-Mignot, Beate Lamers, Peter Lanbeck, Paul Landais, Sybille Landwehr-Kenzel, Vanessa Langemeyer, Thorsten Langer, Petra Lankisch, Nadia Lanz, Manrique de Lara, Eusebia Lara-Villacanas, Lisa Laubenthal, Hans-Jürgen Laws, Loic Le Mignot, Ronan Leahy, Jae-Yun Lee, Andrea Lehmann, Kai Lehmberg, Patricia Lehner, Hans Leibfrit, Leoni Leistner, Petra Lesch, Simon Leutner, Manolis Liatsis, Linda Libai Véghová, Johanna Liebel, Johannes G. Liese, Kotryna Linauskiene, Richard Linde, Martina Linßner, Conrad Ferdinand Lippert, Jiri Litzman, Pilar Llobet, Babacar Lo, Tariq Lodin, Jindrich Lokaj, David Longhino, Hilary Longhurst, Susana Lopes da Silva, Catharina Lorenzen, Vassilios Lougaris, Doris Löw, Annelie Lubatschofski, Mary Lucas, Verena Lutz-Wiegers, Sabine Maaß, Maria Elena Maccari, Anna Macura-Biegun, Vanessa Maerz, Paraskevi Maggina, Hannah Mahrenholz, Sarah Maier, Mounia Makhlouf, Anne Malfroot, Laura Malinauskiene, Wilma Mannhardt-Laakmann, Ania Manson, Felicia Mantkowski, Petra Manzey, Carolina Marasco, Nufar Marcus-Mandelblit, Wolfgang Marg, Gasper Markelj, Laszlo Marodi, José Goncalo Marques, Laura Marques, Karin Marschall, Natalia Martínez, Rafael Martinez de la Ossa Saenz-Lopez, Inmaculada Martinez-Saguer, Baldassarre Martire, Antonio Marzollo, Refiloe Masekela, Katja Masjosthusmann, Tania Nicole Masmas, Nuria Matamoros, Jutta Mattern, Pearl Mau-Asam, Elizabeth McDermott, Nichole McIntosh, Karmen Mesko Meglic, Ruben Meijer, Andrea Meinhardt, Safa Meshaal, Yasmina Messaoud, Björn Meyer, Dirk Meyer-Olson, Romain Micol, Bozena Micoloc, Gudrun Mielke, Cinzia Milito, Tomas Milota, Siraj Misbah, Carolin Mödden, Michael Mohr, Karina Mohrmann, Mostafa Moin, Luis Molinos, Jana Möller, Sarah Möller-Nehring, Henner Morbach, Viviana Moschese, Olga Moser, Despina Moshous, Radoslaw Motkowski, Jayashree Motwani, Nermeen Mouftah Galal, Carmen Müglich, Anna Mukhina, Eva Muller, Christiane Müller, Gabriele Müller, Hedi Müller, Ingo Müller, Thomas Müller, Zoe Müller, Ulf Müller-Ladner, Sarah Müller-Stöver, Esther Münstermann, Núria Murtra Garrell, Moritz Muschaweck, Miriam Mutert, Zohreh Nademi, Paru Naik, Andrea Näke, Andrea Martin Nalda, Gulnara Nasrullayeva, Nora Naumann-Bartsch, Verena Nemitz, Olaf Neth, Andreas Neubauer, Jennifer Neubert, Carla Neumann, Tim Niehues, Mara Niemuth, Chris Nieuwhof, Daniela Nolkemper, Sadia Noorani, Budde Adya Noorlander, Gundula Notheis, Sanam Amelie Nowatsh, Gaelle Obenga, Suheyla Ocak, Mehmet Oker, Peter Olbrich, Anna Olipra, Heymut Omran, Prasad Oommen, Linda Opitz, Jaroslava Orosova, Solveig Oskarsdottir, Hülya Özsahin, Malgorzata Pac, Jana Pachlopnik Schmid, Franco Pandolfi, Efimia Papadopoulou-Alataki, Theodora Papastamatiou, Anna Papatriantafillou-Schmieder, Alba Parra-Martinez, Olga Paschenko, Srdjan Pašić, Jarek Pasnik, Smita Patel, Martin Pavlík, Estela Paz Artal, Anouk Peeters, Sara Branco Pereira da Silva, Ruy Perez-Becker, Marc Perez-Guzman, Markus Perlhagen, Hans-Hartmut Peter, Marin Petrić, Michael Pfreundschuh, Pierre Philippet, Capucine Picard, Barbara Pietrucha, Leonora Pietzsch, Monica Piquer Gibert, Kerstin Pirolt, Alessandro Plebani, Daniel E. Pleguezuelo, Katrin Pollok, Helena Pommerening, Daniela Popihn, Aleksandra Poplonek, Marina Popp, Fulvio Porta, Jan Portegys, Klara Posfay-Barbe, Judith Potjewijd, Sebastian Poulheim, Seraina Prader, Petra Prämassing-Scherzer, Martina Prelog, Johan Prevot, Arthur Price, Timothy Price, Marijke Proesmans, Johan Provot, Federica Pulvirenti, Isabella Quinti, Anna Raab, Anita Rack, Stefan Raffac, Eduardo Ramos Oviedo, Philippe Randrianomenjanahary, Anja Ranohavimparany, Maria Raptaki, Roonaka Rashidzadeh, Margit Rathwallner, Shereen Reda, Nahida Redouane, Frederico S. Regateiro, Janine Reichenbach, Bianca Reimers, Cornelia Reinhardt, Dirk Reinhardt, Anne Reinprecht, Tamara Reiß, Ismail Reisli, Eleonore Renner, Nima Rezaei, Alex Richter, Darko Richter, Nikolaus Peter Rieber, Nadja Rieckehr, Marion Riedel, Heidi Riescher, Johannes Rischewski, Nicole Ristl, Henrike Ritterbusch, Tanja Ritz, Francois Rivier, Jürgen K. Rockstroh, Joachim Roesler, Himatur Rofiah, Elizabeth Rogerson, Elisabeth Rolfes, Beate Roller, Yaryna Romanyshyn, Roberto Rondelli, Fien Roosens, Angela Rösen-Wolff, Valentina Rösler, Johannes Roth, Tobias Rothoeft, Agathe Roubertie, Gesa Rübsam,

Downloaded from http://rupress.org/jhi/article-pdf/1/3/e20250007/1947361/jhi_20250007.pdf by guest on 16 November 2025

Abraham Rutgers, Paul Ryan, Gudrun Sach, Kambis Sadeghi, Ulrike Sahrbacher, Angelika Saidi, Özden Sanal Tezcan, Silvia Sanchez-Ramon, Juan Luis Santos, Ravishankar Sargur, Ihor Savchak, Sinisa Savic, Bernhard Schaaf, Marzena Schaefer, Christina Schäfe, Eva Scharbatke, Ellen Schatorje, Uwe Schauer, Carmen Scheibenbogen, Raphael Scheible, Clemens Scheinecker, Romana Schiller, Beatrice Schilling, Freimut Schilling, Steffi Schlieben, Thilo Schmalbach, Marc Thomas Schmalzing, Pirmin Schmid, Nadine Schmidt, Reinhold Ernst Schmidt, Monika Schmitz, Dominik Schneider, Dominik T. Schneider, Reinhard Schneppenheim, Cathy Scholtes, E.H. Schölvinck, Stefan Schönberger, Stefan Schreiber, Rik Schrijvers, Andrea Schroll, Simone Schruhl, Johanna Schrum, Ralf Schubert, Sebastian Schuh, Ansgar Schulz, Claudia Schulz, Ilka Schulze, Hendrik Schulze-Koops, Ulf Schulze-Sturm, Eva-Maria Schumacher, Elvira Schürmann, Gesine Schürmann, Volker Schuster, Eva Schwaneck, Klaus Schwarz, Tobias Schwarz, Carolynne Schwarze-Zander, Lothar Schweigerer, Wolfgang Schwinger, Anna Sediva, Jörg Seebach, Reinhard Seger, Florian Segerer, Barbara Selle, Suranjith Seneviratne, Hatidje Shabanaj, Anna Shcherbina, Anne Siepelmeyer, Kathrin Siepermann, Anna Simon, Arne Simon, Katja Simon-Klingenstein, Marija Simonovic, Merlin Simsen Baratault, Elena Sindram, Alla Skapenko, Maiken Skarke, Malgorzata Skomska-Pawliszak, Mary Slatter, Julie Smet, Ali Sobh, Bettina Sobik, Georgios Sogkas, Michael Sohm, Xavier Solanich-Moreno, Pere Soler Palacín, Franz Sollinger, Raz Somech, Anja Sonnenschein, Annarosa Soresina, Marijke Sornsakrin, Stavrieta Soura, Sabrina Spaccarotella, Guiseppe Spadaro, Monika Sparber-Sauer, Christof Specker, Carsten Speckmann, Lisa Speidel, Matthaios Speletas, Klaus-Daniel Stachel, Catherine Stadon, Aurélie Stanislas, Cynthia Stapornwongkul, Paulina Staus, Regina Steck, Cathal Steele, Herbert Steffin, Urs Steiner, Sandra Steinmann, Wim Stevens, Martina Stiefel, Sarah Stieger, Sophie Stiehler, Hermann Stimm, Silvia Stojanov, Matthias Stoll, Dominique Stoppa-Lyonnet, Tobias Strapatsas, Gabriele Strauß, Monika Streiter, Riet Strik-Albers, Gaby Strotmann, Héctor Suárez Casado, Jose Luis Subiza, Mikael Sundin, Birgit Süß, Fabienne Sutter, Anna Szaflarska, Monika Szemkus, Hannah Tamary, Sofia Tantou, Michael D. Tarzi, Helga Taschner, Ulf Tedgard, Carla Teixeira, RJM ten Berge, Klaus Tenbrock, Sabine Tester, Ilhan Tezcan, Sonja Thalguter, Julian Thalhammer, Katharina Thoma, Moira Thomas, Fabian Thomczyk, Vojtech Thon, Adrian Thrasher, Patricia Tierney, Nadine Tietsch, Alberto Tommasini, Beate Tönnes, Hans-Peter Tony, Maria Trachana, Carmen Trapp, Lourdes Tricas, Maria Conceicao Trindade Neves, Jordis Trischler, Hugo Ubieto, Anett Uelzen, Annette Uhlmann, Jan Ullrich, Kurt Ullrich, Ekrem Ünal, Gerhard Urbanski, Simon Urschel, Aleksandra Uszynska, Angelo Vacca, N.N. Vaganov, Daniel Vagedes, Stefanie Valicevic, Florence Vallelian, Rachel T. van Beem, Charlotte van Damme, Annick van de Ven, J. Merlijn van den Berg, Michiel van der Flier, J.T. van Dissel, P.M. van Hagen, J.M. van Montfrans, Geoffrey van Ogtrop, Jacqui van Rens, Christel A.M.P. van Riel, Annet van Royen-Kerkhof, G.Th.J. van Well, Antari Vasiliki, Sirje Velbri, Jo Vencken, Alessandro Ventura, François Vermeulen, Christiane Vermylen, Dorothee Viemann, Anja Viereck, Anna Villa, Jeroen Vincke, Lisa Vinnemeier-Laubenthal, Kim Duy Vo Thi, Kristina Vollbach, Alla Volokha, Horst von Bernuth, Philipp von Bismarck, Rebecca Voss, Sandra Voß, Yüksel Vural, Heike Wachuga, Norbert Wagner, Per Wagström, Matthias Wahle, Volker Wahn, Nadine Wapp, Klaus Warnatz, Monika Warneke, Adilia Warris, Jan-Christian Wasmuth, Jean-Blaise Wasserfallen, Angela Wawer, Manfred Weber, Corrie MR Weemaes, Julius Wehrle, Stephan Weidinger, Michael Weiß, Elisabeth Weißbarth-Riedel, Antje Werner, Sara Wessel, Marco Westkemper, Monika Wicher, Lutz Wickmann, Sabine Wiegert, Monique Wiehe, Katharina Wiehler, Lydia Wiesböck, Ewa Wiesik-Szewczyk, Anthony Williams, Beate Winkler, Christel Winkler, Martina Winkler, Melanie Winkler, Uwe Wintergerst, Lukas Wisgrill, Torsten Witte, Helmut Wittkowski, Barbara Wolf, Sandra Wölke, Christina Wolschner, Beata Wolska-Kusnierz, Philip Wood, Sarita Workman, Austen Worth, Michaela Wortmann, Walter Alfred Wuillemin, Nico M. Wulffraat, Katharina Wustrau, Chloé Wyndham-Thomas, Olcay Yegin, Alisan Yildiran, Denise Yilmaz, Patrick Young, Esra Yucel, Milica Zečević, Fred Zepp, Klaus Zetzsche, Rainald Zeuner, Stefan Zielen, Martina Zimmermann, Erić Želimir, Wadih Abou-Chahla, Nathalie Aladjidi, Corinne Armari-Alla, Vincent Barlogis, Sophie Bayart, Stéphane Blanche, Damien Bodet, Bernard Bonnotte, Raphaël Borie, Patrick Boutard, David Boutboul, Claire Briandet, Jean-Paul Brion, Jacques Brouard, Martin Castelle, Pascal Cathebras, Liana Carausu, Emilie Catherinot, Nathalie Cheikh, Morgane Cheminant, Thibault Comont, Louis-Jean Couderc, Pierre Cougoul, Anne Deville, Catherine Devoldere, Eric Dore, Fabienne Dulieu, Isabelle Durieu, Natacha Entz-Werle, Claire Fieschi, Lionel Galicier, Virginie Gandemer, Martine Gardembas, Clément Gourguechon, Bernard Grosbois, Aurélien Guffroy, Corinne Guitton, Gaëlle Guillerm, Mohamed Hamidou, Sophie Haro, Yves Hatchuel, Olivier Hermine, Cyrille Hoarau, Sébastien Humbert, Arnaud Jaccard, Jean-Philippe Jais, Sarah Jannier, Serge Jacquot, Roland Jaussaud, Pierre-Yves Jeandel, Eric Jeziorski, Kamila Kebaïli, Anne-Sophie Korganow, Olivier Lambotte, Fanny Lanternier, Claire Larroche, David Launay, Guillaume Le Guenno, Emmanuelle Le Moigne, Marc Lecuit, Guillaume Lefèvre, Jean-Daniel Lelièvre, Romain Levy, Valérie Li-Thiao-Te, Olivier Lortholary, Luminita Luca, Coralie Mallebranche, Marion Malphettes, Aude Marie-Cardine, Nicolas Martin-Silva, Agathe Masseau, Etienne Merlin, Frédéric Millot, Charline Miot, Floriane Mirgot, Nabila Moussouni, Luc Mouthon, Antoine Néel, Bénédicte Neven, Dalila Nouar, Raphaële Nove-Josserand, Marie Ouachée-Chardin, Anne Pagnier, Catherine Paillard, Marlène Pasquet, Isabelle Pellier, Antoinette Perlat, Christophe Piguet, Dominique Plantaz, Sophie Rivière, Loïc Raffray, Pascal Roblot, Pierre-Simon Rohrlich, Bruno Royer, Valéry Salle, Hélène Salvator, Françoise Sarrot-Reynauld, Amélie Servettaz, Jean-Louis Stephan, Nicolas Schleinitz, Pere Soler Palacín, Felipe Suarez, Laure Swiader, Sophie Taque, Caroline Thomas, Olivier Tournilhac, Caroline Thumerelle, Jean-Pierre Vannier, Jean-François Viallard, Mirjam Voeller, Lisa Wege.

Downloaded from http://rupress.org/jhi/article-pdf/1/3/e20250007/1947361/jhi_20250007.pdf by guest on 16 November 2025
