# Supplementary material for: Inborn errors of immunity: Manifestation, treatment, and outcome—an ESID registry 1994–2024 report on 30,628 patients
Source: J Hum Immun. Author manuscript; Available in PMC 2025 Dec 4. (PMC12674179; doi:10.70962/jhi.20250007)
Supplement: Supplementary file 2 [file NIHMS2107117-supplement-Suppl__Figure_4.pdf]

JHI

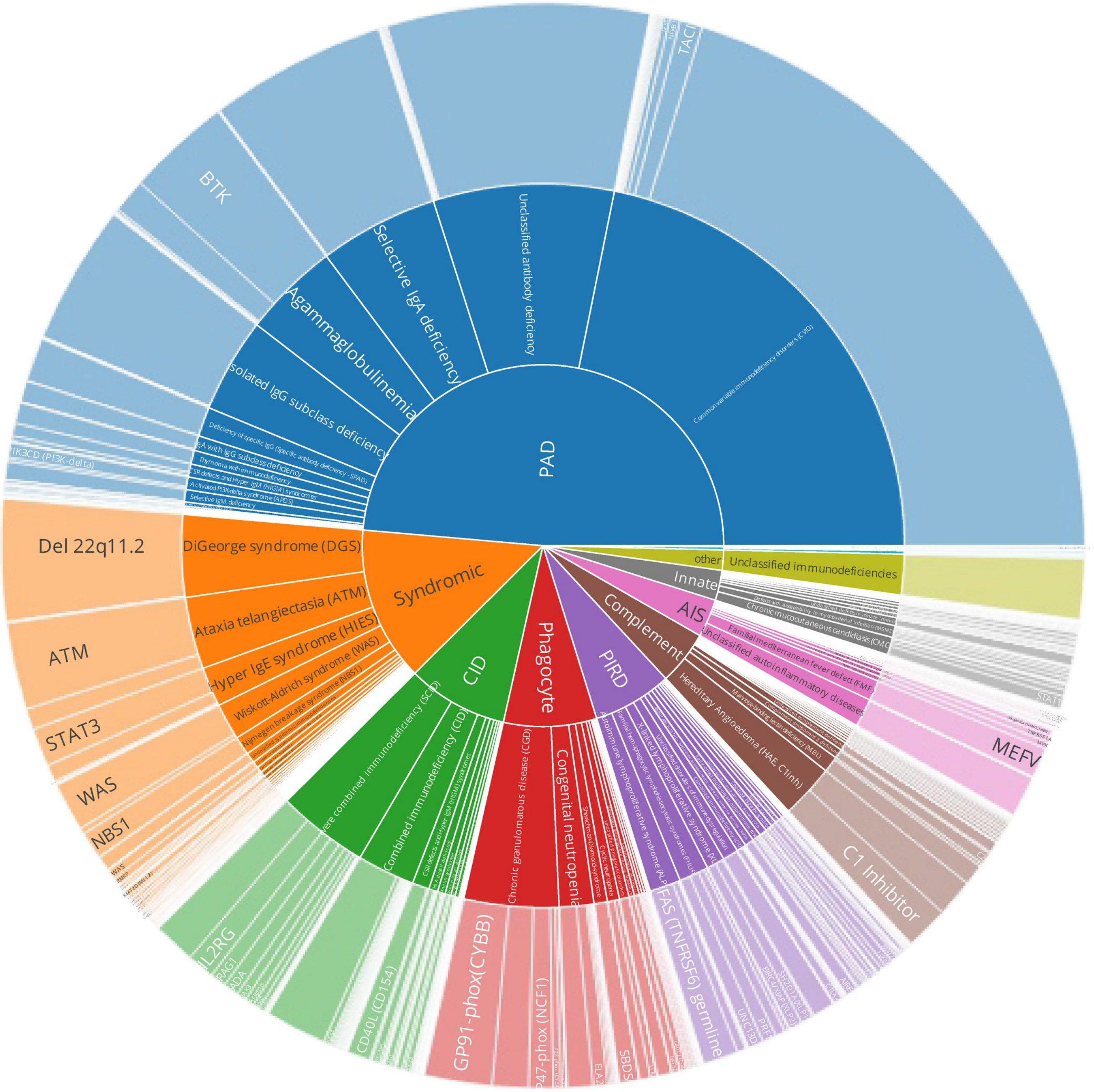

Figure S4. **IEI/PID distribution, nested pie chart for IUIS category, diagnosis, and gene.** This is merely a sketch; please see the interactive version of the figure with all data labels and patient numbers shown on mouse over and click to zoom at https://esid.org/html-pages/Suppl%20Fig%203_ESID_30k_sunburst_PID.html.

Downloaded from http://rupress.org/jhi/article-pdf/1/3/e20250007/1947361/jhi_20250007.pdf by guest on 26 August 2025
