# Supplementary material for: Inborn errors of immunity: Manifestation, treatment, and outcome—an ESID registry 1994–2024 report on 30,628 patients
Source: J Hum Immun. Author manuscript; Available in PMC 2025 Dec 4. (PMC12674179; doi:10.70962/jhi.20250007)
Supplement: Supplementary file 3 [file NIHMS2107117-supplement-Suppl__Figure_5.pdf]

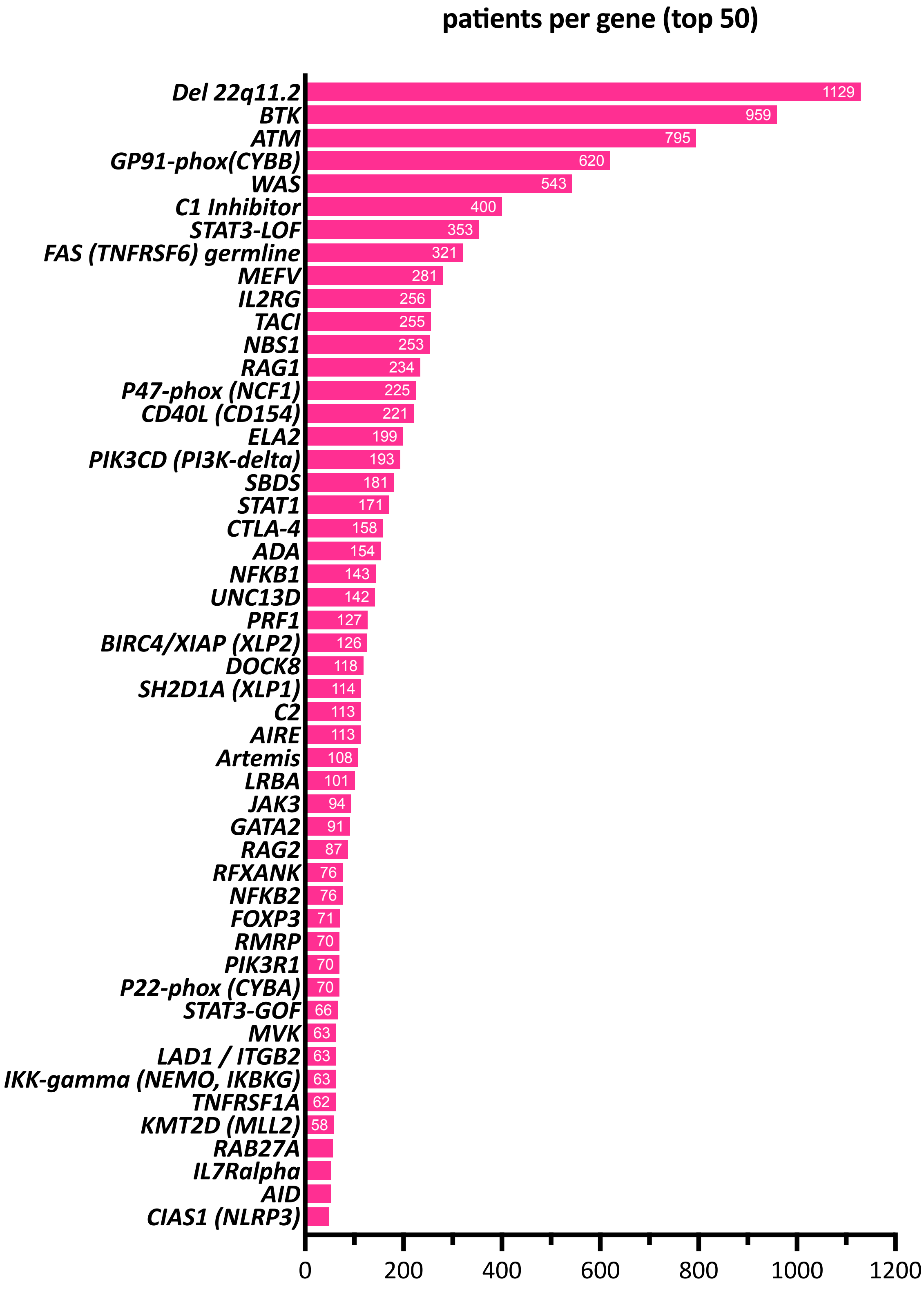

patients per gene (top 50)
Del 22q11.2
1129
BTK
959
ATM
795
GP91-phox(CYBB)
620
WAS
543
C1 Inhibitor
400
STAT3-LOF
353
FAS (TNFRSF6) germline
321
MEFV
281
IL2RG
256
TACI
255
NBS1
253
RAG1
234
P47-phox (NCF1)
225
CD40L (CD154)
221
ELA2
199
PIK3CD (PI3K-delta)
193
SBDS
181
STAT1
171
CTLA-4
158
ADA
154
NFKB1
143
UNC13D
142
PRF1
127
BIRC4/XIAP (XLP2)
126
DOCK8
118
SH2D1A (XLP1)
114
C2
113
AIRE
113
Artemis
108
LRBA
101
JAK3
94
GATA2
91
RAG2
87
RFXANK
76
NFKB2
76
FOXP3
71
RMRP
70
PIK3R1
70
P22-phox (CYBA)
70
STAT3-GOF
66
MVK
63
LAD1 / ITGB2
63
IKK-gamma (NEMO, IKBKG)
63
TNFRSF1A
62
KMT2D (MLL2)
58
RAB27A
IL7Ralpha
AID
CIAS1 (NLRP3)
0
200
400
600
800
1000
1200
