# Supplementary material for: Inborn errors of immunity: Manifestation, treatment, and outcome—an ESID registry 1994–2024 report on 30,628 patients
Source: J Hum Immun. Author manuscript; Available in PMC 2025 Dec 4. (PMC12674179; doi:10.70962/jhi.20250007)
Supplement: Supplementary file 4 [file NIHMS2107117-supplement-Suppl__Figure_6.pdf]

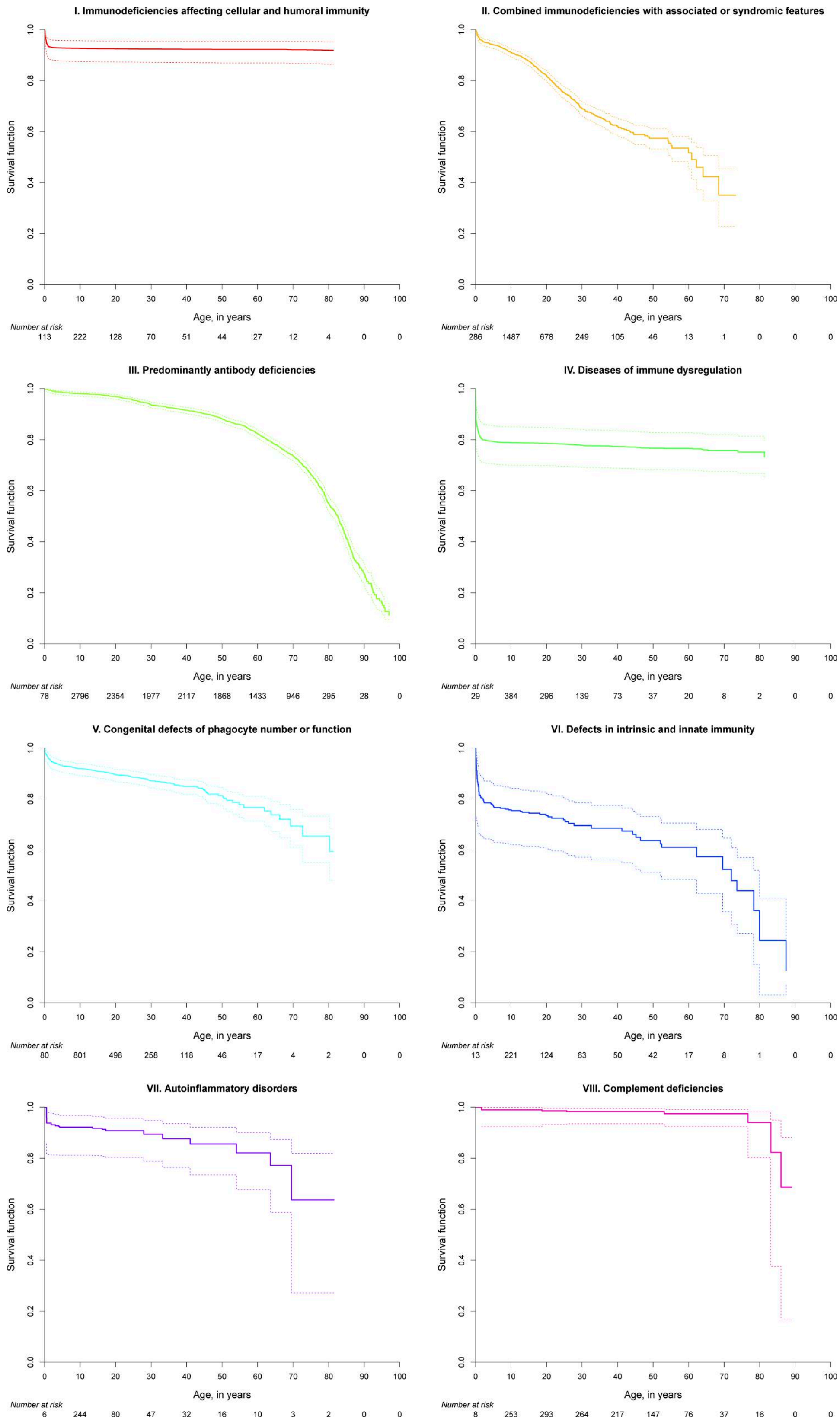

Figure S6. **Survival probabilities of main IUIS categories of IEI/PID and the living status at last news with confidence intervals.** Inverse cumulative incidence curves and confidence intervals as described and referenced above. Start = age at diagnosis, stop = age at last news, and event = living status (0 = censored, 1 = deceased first, 2 = curative therapy first). There were 21,206 patients censored; 1,960 patients who deceased first; and 2,901 patients who had a curative therapy first (not showing deaths after curative therapy); roman numbers refer to the IUIS categories for IEI/PID as listed in Fig. 2 B. Numbers at risk present patients in the ESID-R from their age at diagnosis onward until definitive/curative treatment or death.

Downloaded from http://rupress.org/jhi/article-pdf/1/3/e20250007/1947361/jhi_20250007.pdf by guest on 26 August 2025
