# Supplementary material for: Inborn errors of immunity: Manifestation, treatment, and outcome—an ESID registry 1994–2024 report on 30,628 patients
Source: J Hum Immun. Author manuscript; Available in PMC 2025 Dec 4. (PMC12674179; doi:10.70962/jhi.20250007)
Supplement: Supplementary file 5 [file NIHMS2107117-supplement-Suppl__Figure_7.pdf]

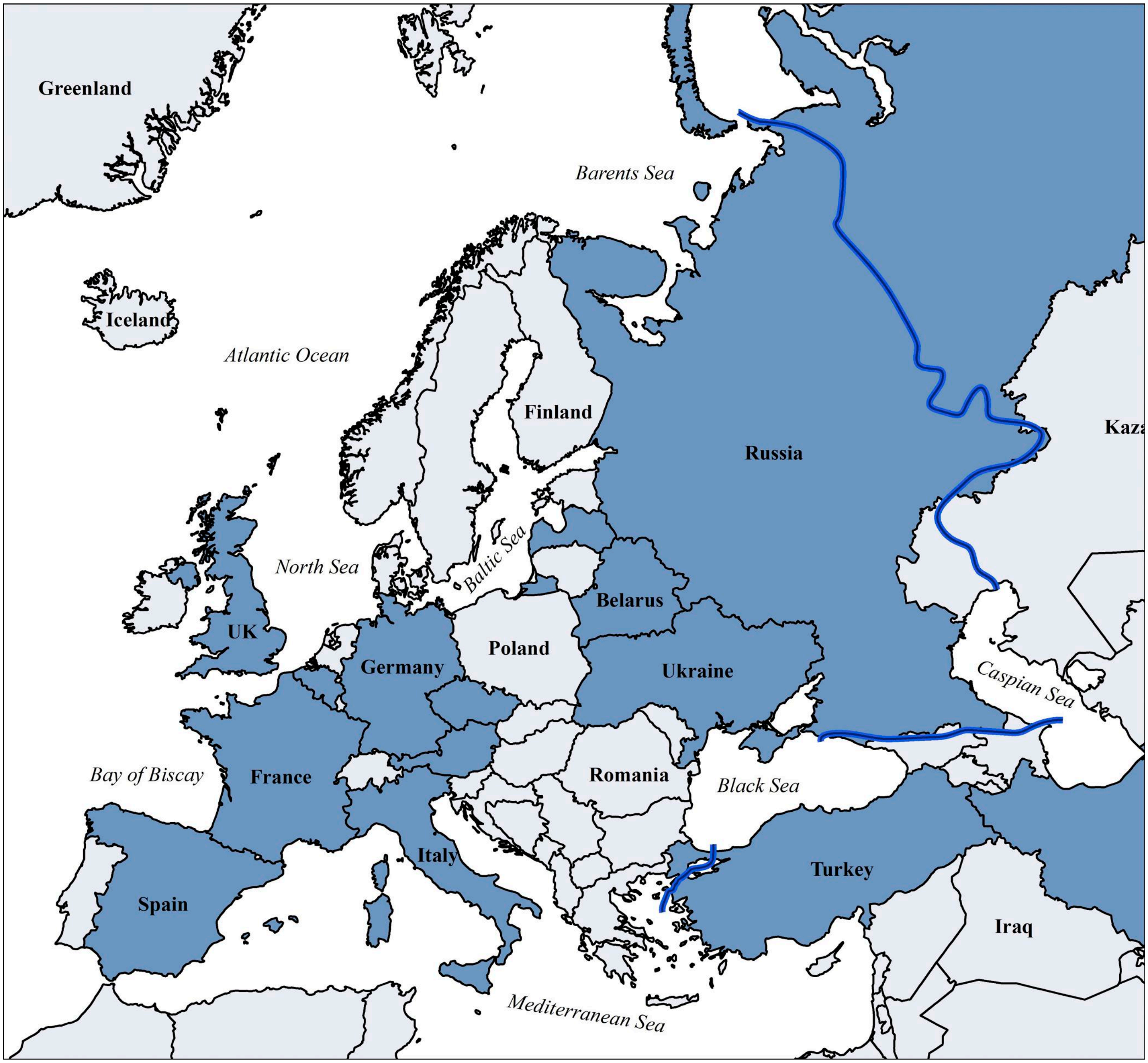

Figure S7. **Countries with national patient registries or national sub-registries within the ESID-R according to the 2024 ESID survey (all participating IEI/PID centers worldwide are listed in** Table S5**).** Blue line: approximate boundary of geographical Europe.

Downloaded from http://rupress.org/jhi/article-pdf/1/3/e20250007/1947361/jhi_20250007.pdf by guest on 26 August 2025
