# Supplementary material for: Inborn errors of immunity: Manifestation, treatment, and outcome—an ESID registry 1994–2024 report on 30,628 patients
Source: J Hum Immun. Author manuscript; Available in PMC 2025 Dec 4. (PMC12674179; doi:10.70962/jhi.20250007)
Supplement: Supplementary file 6 [file NIHMS2107117-supplement-Supplementary_Material.pdf]

JHI

Supplemental material

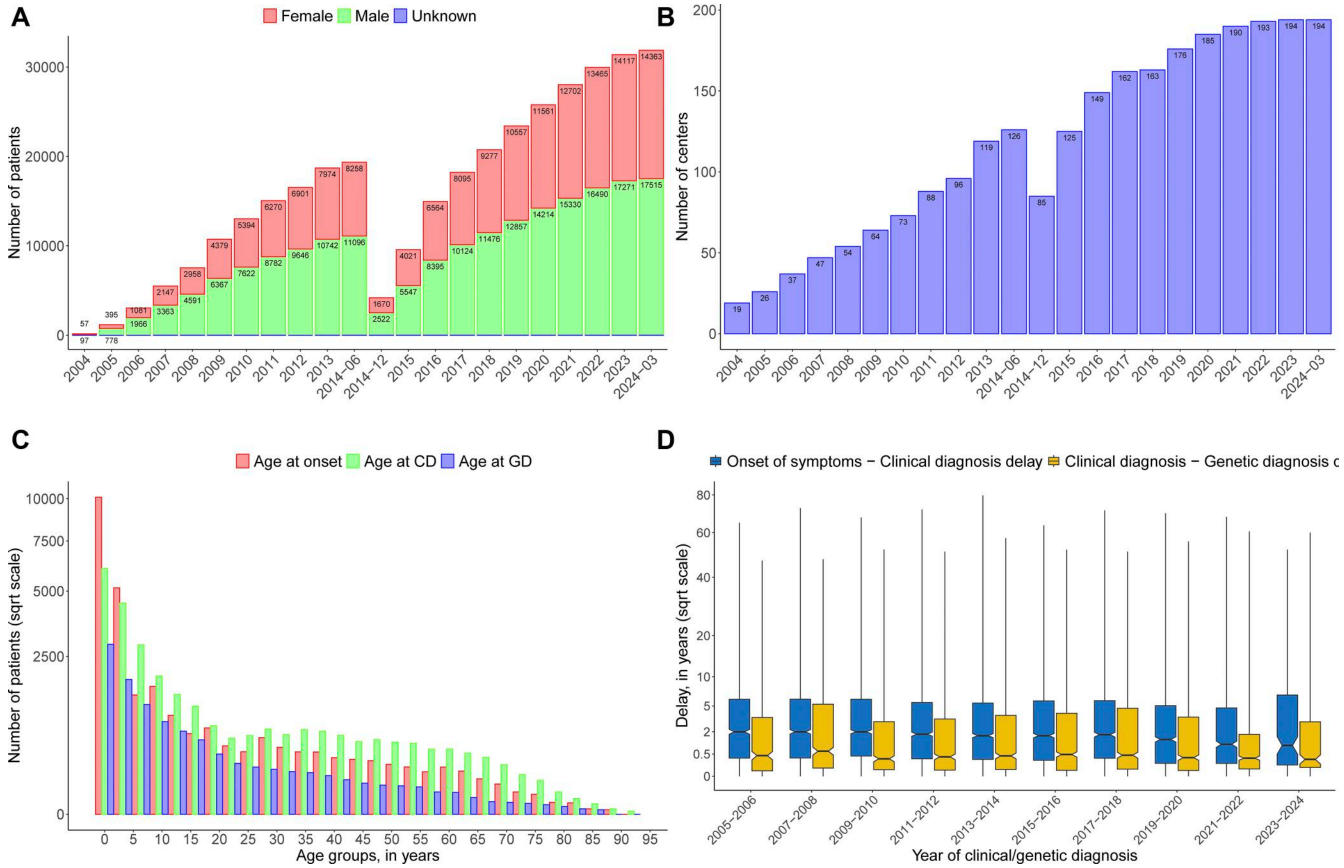

Figure S1. **The history of patient inclusion into the ESID registry over the last 20 years. (A, upper left panel)** Cumulative number of patients registered in the ESID-R. The total number shown here is 31,889 patients (male *n* = 17,515; female *n* = 14,363; unknown *n* = 11), before exclusion of those discharged (*n* = 538), those with secondary immunodeficiency (*n* = 417), and those without a definitive diagnosis of IEI/PID (*n* = 306). Data are shown for the online registry only. Data from the very first version (hardcopy) were not transferred to the first online registry but newly entered. According to "The Source" (https://esid.org/wp-content/uploads/2024/03/ESID_TheSource_2003.pdf), the total number in 2003 was 9,707 patients. **(B, upper right)** Cumulative number of ESID-R participating centers. **(C, lower left)** Age at onset of IEI or PID. Age groups of age at disease onset, clinical diagnosis (CD) of IEI/PID, and of genetic diagnosis (GD) are shown. **(D, lower right)** Diagnostic delay (in years from first manifestation to clinical diagnosis and from clinical diagnosis to genetic diagnosis) of patients diagnosed over the last 20 years in the ESID-R. The x axis shows the year of clinical/genetic diagnosis in 2-year intervals. The blue boxes represent the delay between onset of symptoms and clinical diagnosis (blue) and between clinical and genetic diagnosis (orange); the y axis shows the delay in years: median with 95% confidence interval (notch size of boxes), interquartile range (boxes), and range (min-max, whiskers).

Downloaded from http://rupress.org/jhi/article-pdf/1/3/e20250007/1947361/jhi_20250007.pdf by guest on 17 July 2025

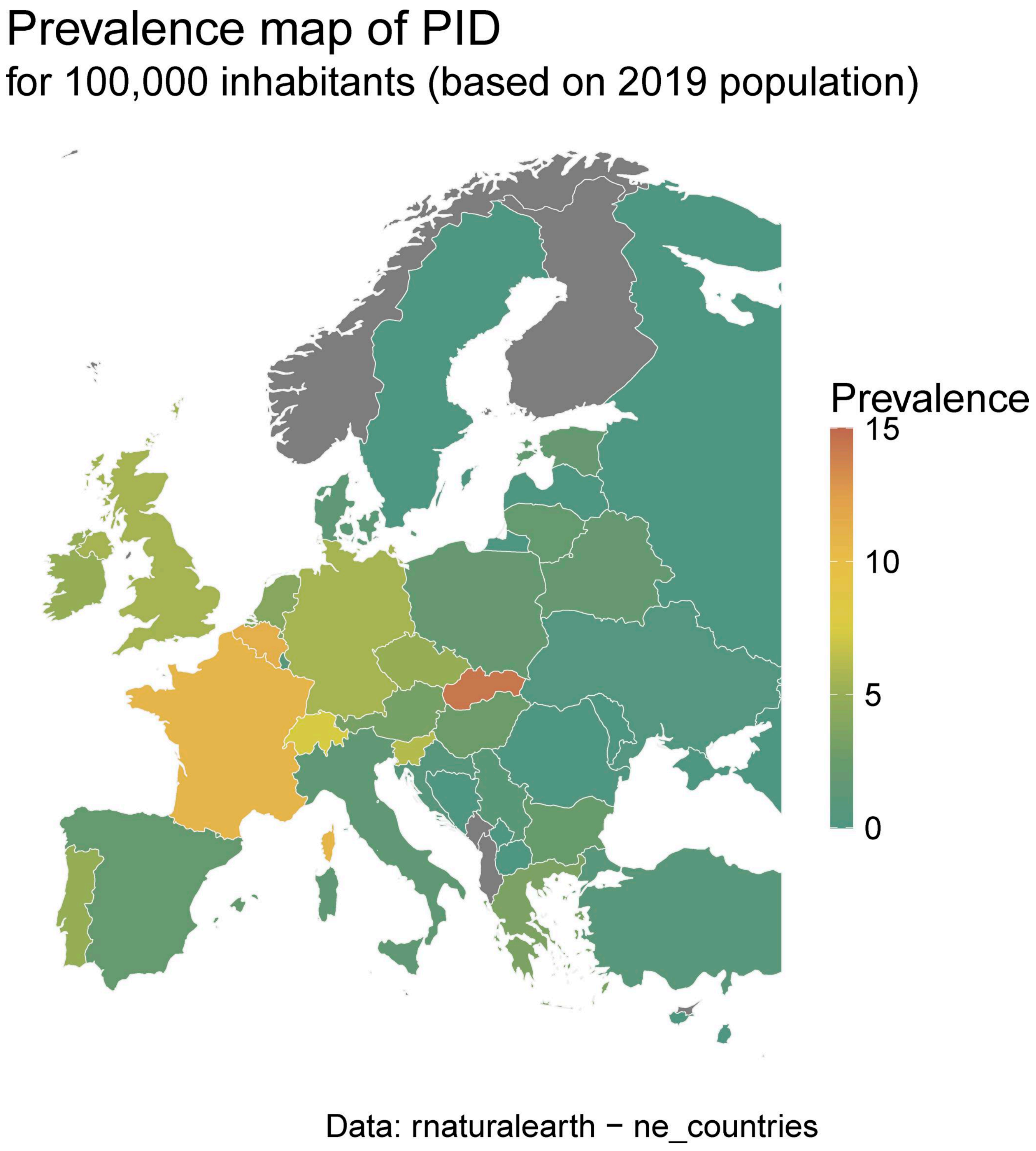

Figure S2. **IEI/PID minimal prevalence map according to ESID-R patient numbers and the 2019 population (same data as in** Table S1**).**

Downloaded from http://rupress.org/jhi/article-pdf/1/3/e20250007/1947361/jhi_20250007.pdf by guest on 17 July 2025

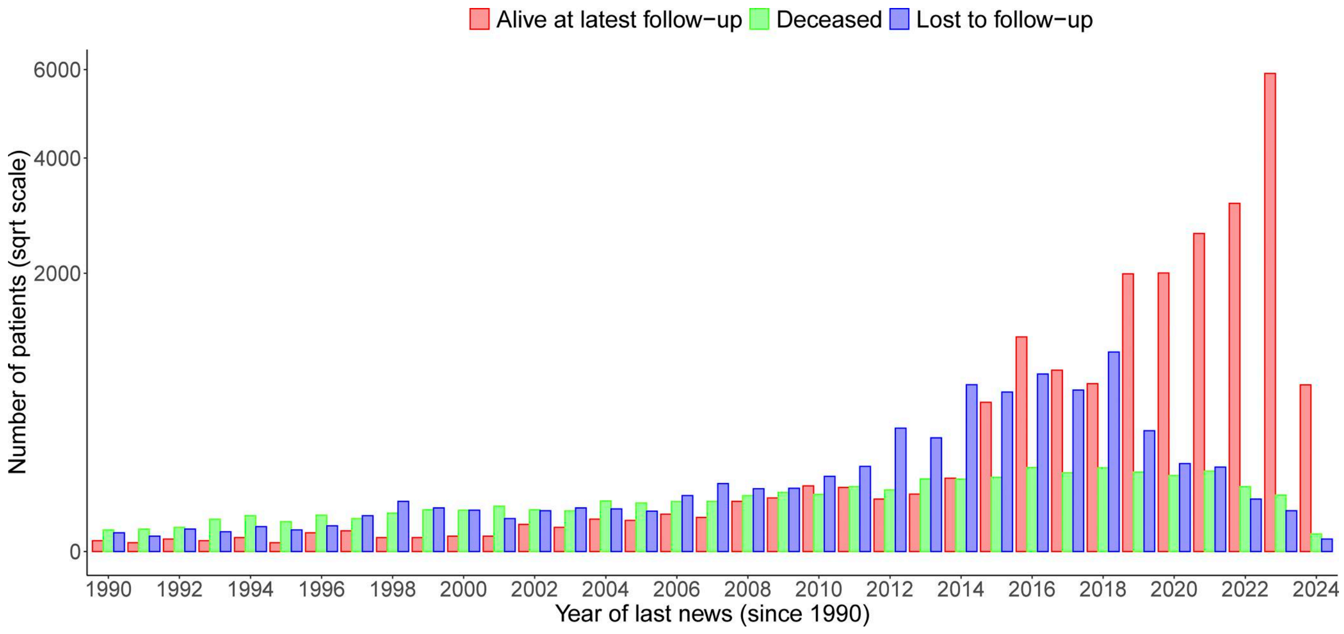

Figure S3. **Living status of patients at the year of last news in the ESID-R captured until March 2024.** “Last news” is the date when the patient was last seen or his/her condition was reported (by telephone or medical report). It is not the documentation date. Together, these data reflect the overall follow-up rate and living status of patients up to the time point of data closure in March 2024.

Downloaded from http://rupress.org/jhi/article-pdf/1/3/e20250007/1947361/jhi_20250007.pdf by guest on 17 July 2025

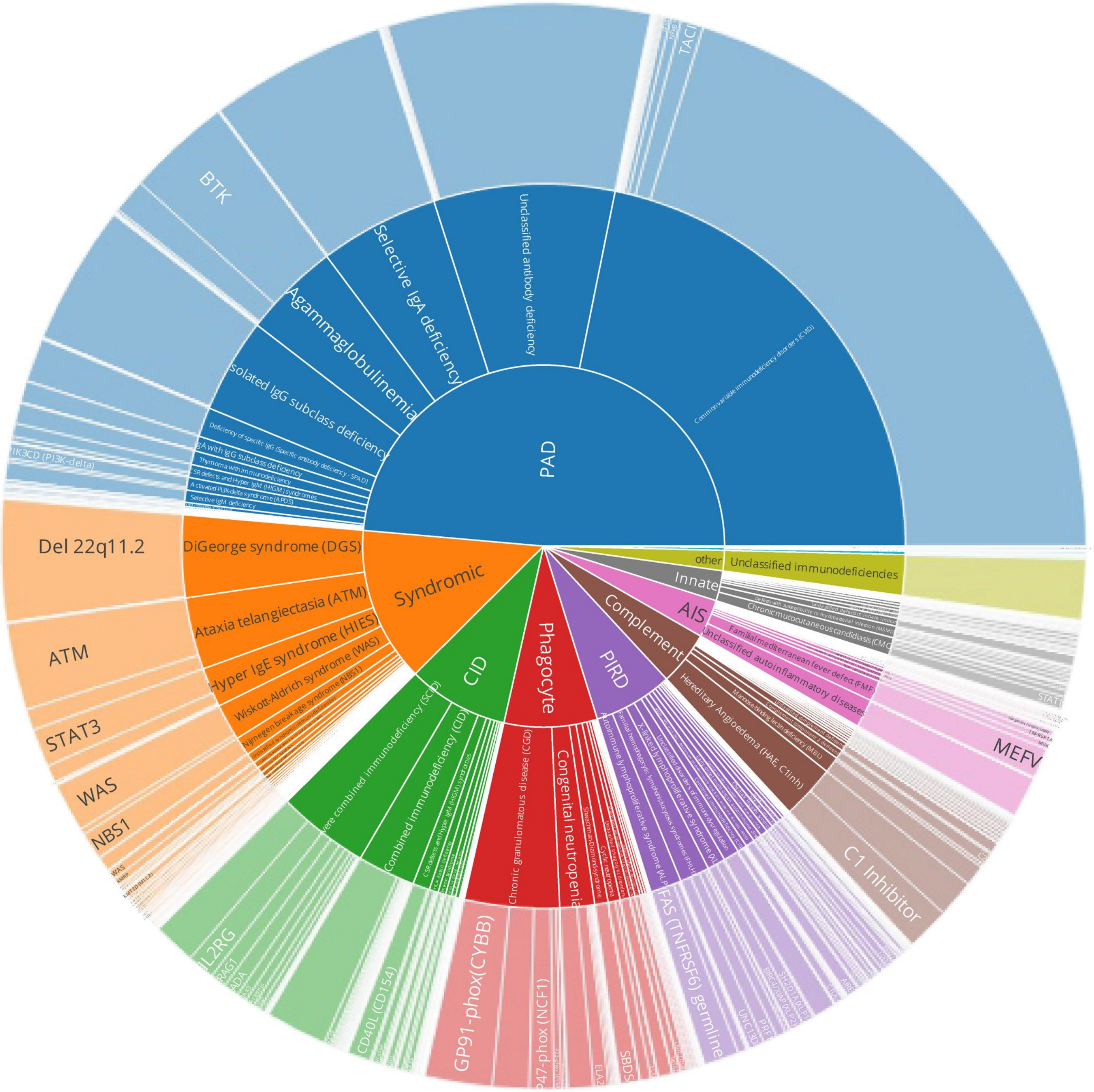

Figure S4. **IEI/PID distribution, nested pie chart for IUIS category, diagnosis, and gene.** This is merely a sketch; please see the interactive version of the figure with all data labels and patient numbers shown on mouse over and click to zoom at https://esid.org/html-pages/Suppl%20Fig%203_ESID_30k_sunburst_PID.html.

Downloaded from http://rupress.org/jhi/article-pdf/1/3/e20250007/1947361/jhi_20250007.pdf by guest on 17 July 2025

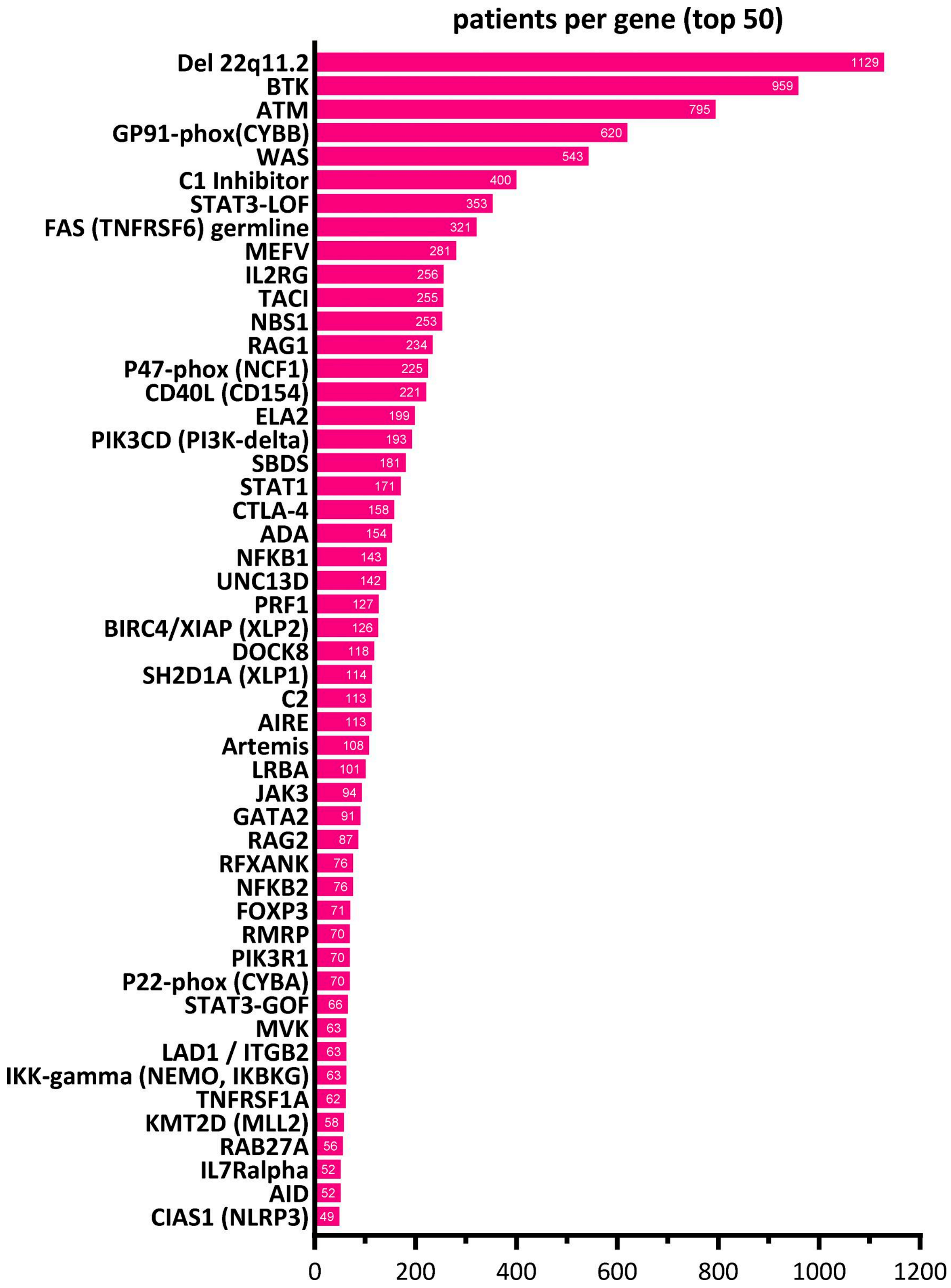

Figure S5. **Top 50 genetic diagnoses of IEI/PID patients recorded in the ESID-R.** The top 50 genes mutated in ESID-R patients with monogenic IEI/PID are shown with respective patient numbers in descending order.

Downloaded from http://rupress.org/jhi/article-pdf/1/3/e20250007/1947361/jhi_20250007.pdf by guest on 17 July 2025

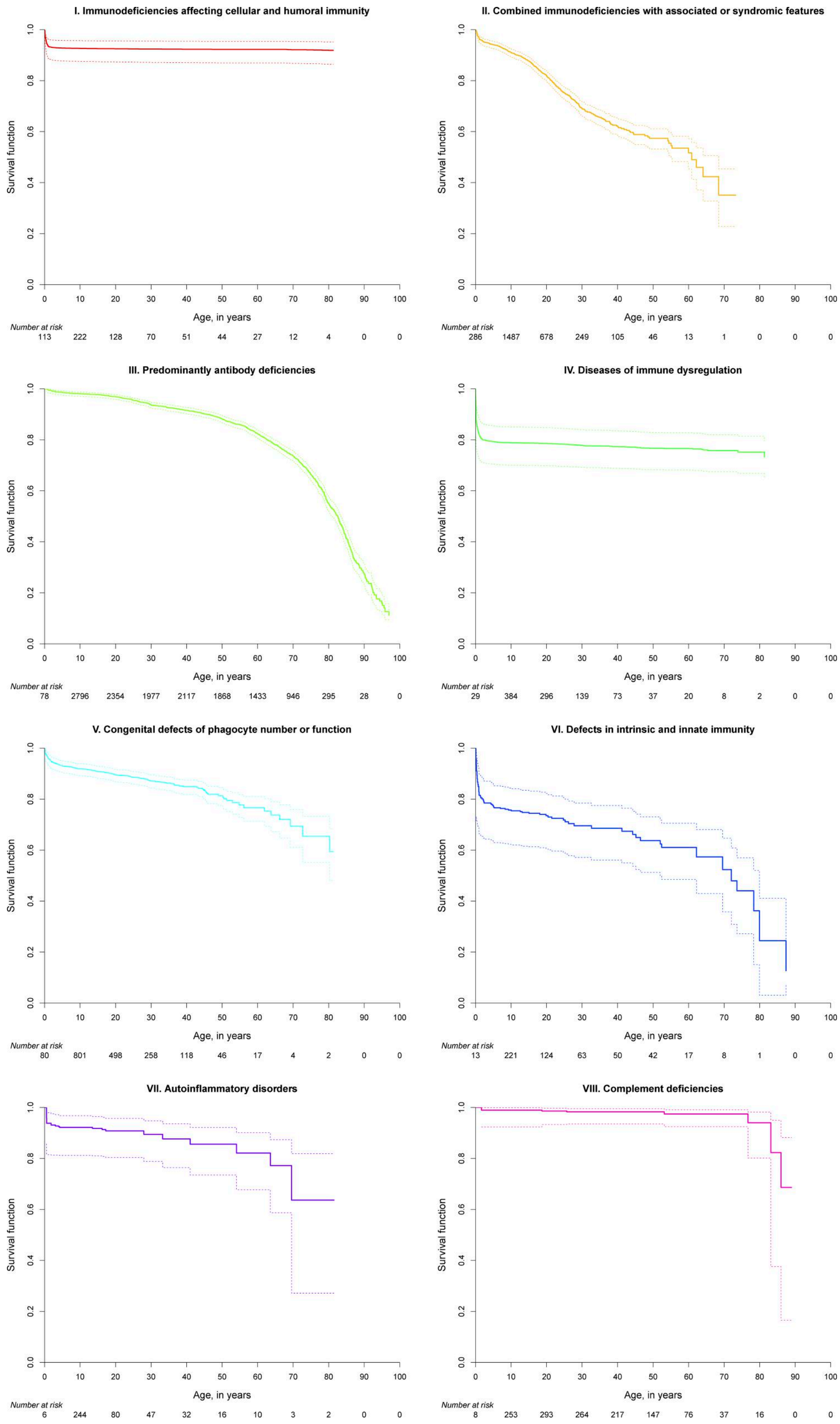

Figure S6. **Survival probabilities of main IUIS categories of IEI/PID and the living status at last news with confidence intervals.** Inverse cumulative incidence curves and confidence intervals as described and referenced above. Start = age at diagnosis, stop = age at last news, and event = living status (0 = censored, 1 = deceased first, 2 = curative therapy first). There were 21,206 patients censored; 1,960 patients who deceased first; and 2,901 patients who had a curative therapy first (not showing deaths after curative therapy); roman numbers refer to the IUIS categories for IEI/PID as listed in Fig. 2 B. Numbers at risk present patients in the ESID-R from their age at diagnosis onward until definitive/curative treatment or death.

Downloaded from http://rupress.org/jhi/article-pdf/1/3/e20250007/1947361/jhi_20250007.pdf by guest on 17 July 2025

JHI

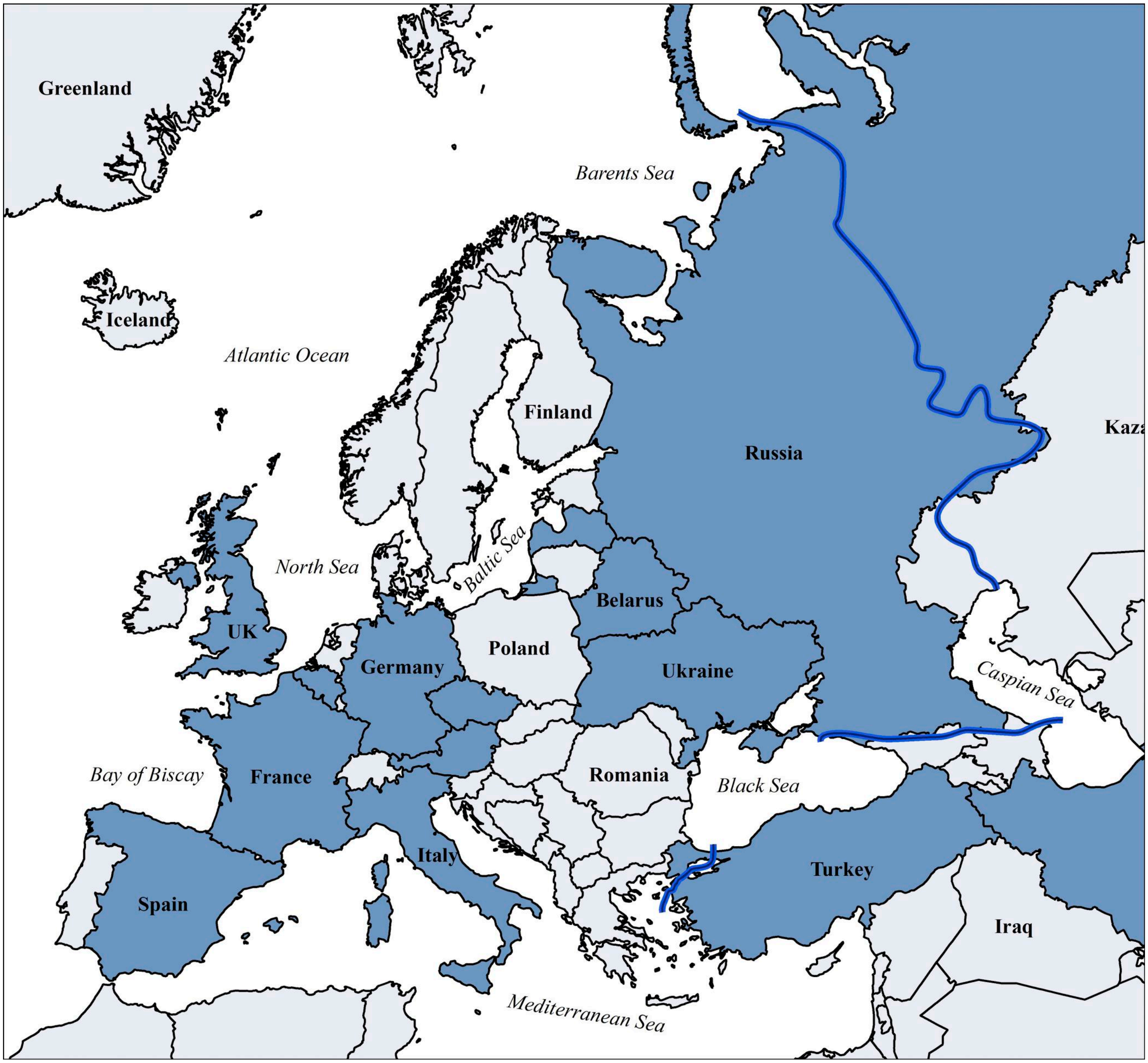

Figure S7. **Countries with national patient registries or national sub-registries within the ESID-R according to the 2024 ESID survey (all participating IEI/PID centers worldwide are listed in** Table S5**).** Blue line: approximate boundary of geographical Europe.

Downloaded from http://rupress.org/jhi/article-pdf/1/3/e20250007/1947361/jhi_20250007.pdf by guest on 17 July 2025

Historical evolution of the ESID-R, objectives, structure, and content

The transition from the first hard-copy-based to the first online version of the ESID-R was headed by Bodo Grimbacher, Freiburg, Germany, with the help of Barbara Frisch and Viviane Knerr, as well as programmers Dominik Veit and Stephan Rusch, in 2000–2004. The biggest hurdles faced during this first restructuration were ethical considerations, data protection, and obtaining industry funding. The second overhaul was conducted by Stephan Ehl, Freiburg, and programmers Benjamin Gathmann and Stephan Rusch in 2014 and focused on content restructuration. The ESID-R steering group, led by the elected chairperson of the ESID-R Working Party, oversees all aspects of the evolution of the registry, including the design, operations, financing, and setup of dedicated studies.

Specific objectives of the ESID-R include.

(1) Collection of epidemiological data: The registry contains basic information on patients with IEI, including their medical history, diagnosis, genetics, treatment, and disease progression. Data on the prevalence of these rare diseases and the associated health burden are important for political decision-makers.
(2) Analysis and research: By analyzing the collected data on these rare diseases through multicenter collaboration, researchers can gain new insights into the pathophysiology and treatment of primary immunodeficiencies.
(3) Support of clinical studies and trials: Registry data can serve as the basis for clinical studies or trials aimed at developing new therapies, improving the treatment of patients affected by those diseases, and for post-marketing surveillance studies of new drugs and medicinal products.
(4) Improving patient care: Through the analyses and publication of registry data, the registry helps improve IEI patient care by providing physicians and healthcare providers/decision-makers with access to up-to-date information and practices.

Both versions of the ESID-R operating from 2004–2014 and 2014–2024 had the following key features (33):

(1) Database architecture: The ESID-R utilizes a sophisticated database architecture to manage the vast amount of data collected from patients. This database is designed to be scalable, ensuring it can handle the increasing volume of patient records and research findings over time.
(2) Data security: Ensuring the security and privacy of patient data is paramount. The ESID-R implements stringent security measures to protect sensitive information, including encryption protocols and access controls.
(3) Web-based platform: The ESID-R is accessed through a web-based platform, allowing authorized users to input and access data remotely. This platform is user-friendly and intuitive, facilitating seamless data entry and retrieval processes. Only an up-to-date web browser and stable internet connection are required to access the system. No additional on-site software needs to be installed/maintained at the documentation centers.
(4) Framework and technologies: The ESID-R is built using a combination of programming languages, frameworks, and technologies suited to its specific requirements. Common technologies include SQL databases for data storage, web development frameworks for the user interface, and data analysis tools for reporting and research purposes.

The three-level content structure

The old version of the online ESID-R (2004–2014) was closed for documentation on June 25, 2014. During the first main revision of the online registry in 2014, a three-level structure was implemented. Level 1 addresses the task of the registry as a simple epidemiological tool. To achieve patient registration by as many centers as possible, the dataset is kept short and simple such that caregivers who know the patient well can fill in the data without consulting the patient chart in much detail. It contains basic epidemiological information, information about main categories of symptoms at initial presentation, and information about some key therapies (immunoglobulins, HSCT, and GT). The yearly update is restricted to survival and implementation of major therapies. The level 1 registration also serves to identify patients for additional in-depth studies, including interventional trials. Level 2 was designed for long-term studies on certain diseases or disease groups without a predefined end. Level 2 datasets allow registration of a limited number of clinical manifestations and lab values that are provided on a yearly basis. It was particularly designed to capture longitudinal data in disease groups, for which the molecular basis has not been fully resolved (such as CVID or specific antibody deficiencies). Again, the datasets are kept simple to limit the burden of registration. Level 3 was designed for studies on particular genetically defined diseases based on a study protocol with a defined endpoint. The level 3 dataset can contain very detailed information, including grading of symptoms, more sophisticated lab values, detailed treatment information, and quality of life data. Registration intervals are at least yearly. Level 3 studies usually offer financial support for the documenting centers.

The level 2 and 3 projects or "sub-studies" are designed by independent research groups. To apply for implementation of a study in the ESID-R, a statistical evaluation plan, a time and budget forecast, need to be defined in a standardized study proposal, which is assessed for feasibility and to avoid redundancy or competing interests by the registry steering committee and the elected chairperson in regular meetings. There should also be a financial contribution from the principal investigator or research institution to (at least partly) cover the costs associated with the setup, coordination, data maintenance, and analyses of these registry sub-studies (details on ESID website, fee structure). When signing the informed consent, patients may choose to have their anonymized data be included either in sub-studies of academic research groups or of pharmaceutical industry–sponsored consortia, or none of them.

Downloaded from http://rupress.org/jhi/article-pdf/1/3/e20250007/1947361/jhi_20250007.pdf by guest on 17 July 2025

Supplemental methods

The ESID-R operates based on a technical infrastructure designed to securely receive, store, and enable the analysis of data related to IEI. These data cover, for example, various aspects of the disease, including genetic mutations, clinical symptoms, laboratory test results, imaging findings, and treatment outcomes. Patients with IEI are registered in the web-based database when their disease data are submitted by an authorized physician or healthcare professional or documenting personnel. Centers or sites where patients are cared for are typically university clinics/hospitals and have a data transfer and general data protection regulation-compliant handling agreement and a local IRB approval in place. The data are de-identified and kept strictly confidential to protect patient privacy.

During the evolution of the registry, it was possible to enter diseases (e.g., LRBA) in multiple categories depending on the development of reported phenotypes and assignment within the IUIS classification. Only with the last update in 2024 a "genetic hierarchy" for categorizing diseases was introduced.

Different measures are applied to avoid duplicates. Firstly, for centers that use the "personalized version" of the registry, where identifying data is kept on a separate server, the pseudonymization tool (Mainzelliste) creates a notification that this (or a similar) patient seems to have been registered before. The system thereby encourages the documenting person to investigate further before proceeding with the creation of a new patient documentation. This option is not available for centers that enter pseudonyms directly. Secondly, persons who sign the consent (patients or parents, next of kin) are always asked whether they have signed a similar consent before. These measures reduce the likelihood of double registrations in the first place. Thirdly, for patients who are transferred from pediatric to adult centers or who move from one region or country to another, usually the caring hospital is aware of the site where the follow-up is done in the future, and, if this is an ESID-R documenting center, the patient data can be reassigned to the new center internally, and follow-up for the adult or relocated patient can be continued in the same documentation under the new center's name.

The Kaplan–Meier curves were generated as described (32). The Kaplan–Meier is done using the start and stop notation to take care of the left truncation effect. Start = age at diagnosis, stop = age at last news, and event = living status (0 = censored, 1 = deceased first, 2 = curative therapy first). There were 21,206 patients censored; 1,960 patients who deceased first; and 2,901 patients who had a curative therapy first.

Supplemental results and discussion

NBS and longitudinal patient documentation in the ESID-R. The ESID-R data structure does not (yet) allow an analysis of improved diagnostic delay and survival in patients diagnosed with SCID in the ESID-R in the NBS era (Fig. S1 C). Firstly, NBS for SCID has been performed sequentially over the last decade, but is still not generally available; and secondly, collecting the intended annual follow-up information may cause a system-inherent delay, including underreporting of SCID patients following definitive therapy (or death), before their data are stored in the ESID-R. The ESID-R Working Party continues to face significant challenges while aiming to integrate data collected over a patient's entire disease course into a single registry, as this ranges from NBS or clinical diagnosis, through different clinical events, such as a form of definitive therapy, and/or chronic treatment, and/or their optional participation in observational sub-studies and on to a cure or until death. The living status at last news from patient is shown in Fig. S3.

The ESID-R in the international landscape of registries for patients with rare immunological diseases. To depict the current situation of the global IEI registry landscape and participants' motivations, we searched PubMed, communicated with representatives of sister societies around the globe, and performed an online survey in 2024. Of 533 publications retrieved by searching for primary immunodeficiency patient registry and exclusion of disease-specific or other secondary sub-studies, the remaining publications showed that a vast majority of IEI/PID registries are national epidemiological databases with a limited temporal and geographical scope regarding longitudinal patient data (32, 33, 34, 35, 36, 37, 38, 39, 40, 41, 42, 43, 44, 45, 46, 47, 48, 49, 50, 51). They, naturally, contain a substantially smaller number of patient datasets than the continental ESID-R, which also includes data from patients of some neighboring countries. As such, they are valuable and necessary platforms for the connection of healthcare providers and clinical experts, as well as for health politicians and economists. A relatively recently published overview of the global registry landscape and the burden of IEI/PID from 80 countries evaluated the distribution of IEI/PID diagnoses and other factors, such as the diagnostic delay or immunoglobulin treatment in relation to the countries' resources and societal factors, such as the rate of consanguinity (12). These data show that the key drivers to implement and maintain an IEI/PID patient registry range from health policy, warranting access to expert care and modern treatments, over patient advocacy, to clinical, biological, and pharmaceutical research and development. Importantly, International Patient Organisation for Primary Immunodeficiencies, the global patient organization for IEI, has identified the existence of registries as one of the six key principles of care as part of its PID LIFE Index, a tool to measure the quality of IEI/PID healthcare in a country (52). To analyze the policy of patient inclusion and obtain feedback from documenting physicians or study nurses, we designed a survey distributed through ESID in 2024. A total of 43 physicians from 29 different European and non-European countries participated in this survey (Table S5). 70% of respondents (30/43) said they would exclusively register their patients into the ESID-R; 30% of the survey respondents (13/43) said they would also enter patient data into one of the following disease-specific (other than general IEI/PID) registries: Eurofever, Severe Chronic Neutropenia International Registry, Juvenile Inflammatory Rheumatism Cohort, and the registry of the EBMT/Center for International Blood and Marrow

Downloaded from http://rupress.org/jhi/article-pdf/1/3/e20250007/1947361/jhi_20250007.pdf by guest on 17 July 2025

Transplant Research. Motivations for entering data into the ESID-R included collaborative research (88%), networking (84%), taking part in academic or pharmaceutical studies (84% and 72%), and improvements in patient care (72%) and for IEI education purposes. Reasons given for not entering patient data into the ESID-R were lack of personnel (84%), legal challenges regarding data transfer (37%), no obvious local benefit (23%), and concerns about small countries being underrepresented (14%). Survey participants stated that they would like a simplified version of the patient consent form, easy interoperability between national registries and the ESID-R, and grant incentives for entering patient data, especially if these were needed at a higher level of granularity.

HPO in IEI/PID and potential benefit of integrating HPO into the ESID-R. The HPO comprehensively organizes and defines the phenotypic features of human disease, supporting genomic and phenotypic analyses through semantic similarity and machine learning algorithms (31). Currently, the HPO terminology for IEI/PID still needs to be further developed with help of the community of clinical immunologists and researchers focusing on IEI/PID, i.e., new HPO terms for relevant phenotypic features to be added, and existing HPO terms to be revised, such that the definitions and hierarchical placement in the HPO reflect current knowledge (26). The HPO can be used with the Global Alliance for Genomics and Health Phenopacket Schema to represent not only phenotypic features but also measurements, biopsies, and treatments over the entire time course of a disease (53). Collections of phenopackets may support translational research in immunology by providing standardized input data for software programs designed to identify genotype–phenotype correlations and characterize disease subtypes (54) (*Preprint*). Integrating the HPO terminology into the ESID-R would inspire studies within or across IUIS categories, especially in monogenic entities. To make full use of the HPO, better systems for simple but comprehensive and accurate data entry are needed.

Provided online are Table S1, Table S2, Table S3, Table S4, and Table S5. Table S1 shows the contributing countries, sorted by the calculated prevalence per 100,000 inhabitants. Table S2 shows the underlying and main causes of death. Table S3 shows the therapy. Table S4 shows the sub-analysis of IEI/PID diagnoses in patients who died before the age of 5 years in two subcategories of IEI/PID. Table S5 shows the institutions of participants of the 2024 ESID-R survey on the international IEI/PID registry landscape.

Downloaded from http://rupress.org/jhi/article-pdf/1/3/e20250007/1947361/jhi_20250007.pdf by guest on 17 July 2025

Appendix

ESID-R Working Party members: Dalia Abd Elaziz, Sohilla Lofty M. Abdelkader, Avigaelle Abitbol, Hassan Abolhassani, Lalash Abrahim, Mohamed Abuzakouk, Nabila Achir Moussouni, Veronica Afonso, Philipp Agyeman, Martina Ahlmann, Alessandro Aiuti, Abla Akl, Güzide Aksu, Kim Albers, Meda Diana Alecsandru, Olga Aleinikova, Svetlana Aleshkevich, Luis Allende, Zoe Allwood, Manrique de Lara Laia Alsina, Daniela Ambrosch-Barsoumian, Lisa Ameshofer, Kenza Amour, Evrim Anadol, Ariharan Ananthachagaran, Chantal Andriamanga, Julia Andris, Karin Andritschke, Federica Angelini, Tobias Ankermann, Katrin Apel, Siamak Arami, Ömür Ardeniz, Peter Arkwright, Karina Arnold, Rudolf Ascherl, Najla Assam, Nurit Assia-Batzir, Faranaz Atschekzei, Sybille Aumann, Volker Aumann, Magnus Aurivillius, Bernd Ausserer, Tadej Avcin, Sezin Aydemir, Emel Aygören-Pürsün, Cagdas Ayvaz Deniz, Chiara Azzari, Perrine Bach, Sophie Bachmann, Peter Bader, Shahrzad Bakhtiar, Renate Bancé, Catherine Bangs, Katerina Barfusz, Safa Baris, B. Barlan Isil, Michaela Bartsch, Helge Baumann, Ulrich Baumann, Veronika Baumeister, Helen Baxendale, Suzanne Bazen, Beatrice Beaurain, Julien Beauté, Mounia Bechar-Makhloufi, Norbert Beck, Brigitta Becker, Christian Becker, Uta Behrends, Renata Beider, Rita Beier, Amel Belkacem, Luisa Belke, Sven Bellert, Bernd H. Belohradsky, Lilia Ben Slama, Aouatef Ben-Bouzid, Vincent Benoît, Thamila Berdous-Sahed, Jan Bergils, Anna Berglöf, Peter Bergman, Katarzyna Bernat-Sitarz, Jolanta Bernatoniene, Ewa Bernatowska, Benedikt Bernbeck, Claire Bethune, Kai Beuckmann, Malini Bhole, Anika-Kerstin Biegner, Stefan Bielack, Kirsten Bienemann, Arndt Bigl, Amélie Bigorgne, Marc Bijl, Nadine Binder, Michaela Bitzenhofer-Grüber, Geraldine Blanchard Rohner, Dagmar Blank, Claudia Blattmann, Julia Blau, Audra Blaziene, Stefan Blazina, Roswitha Blume, Barbara Boardman, Sebastian Bode, Jaap-Jan Boelens, Christoph Boesecke, Delfien Bogaert, Johannes Bogner, Nadezda Bohynikova, Claire Booth, Victoria Bordon, Arndt Borkhardt, Melanie Börries, Michael Borte, Stephan Borte, Lukas Bossaller, Madeleine Bossard, Jeannet Botros, Soraya Boucherit, Jeannette Boutros, Oksana Boyarchuk, Onur Boyman, Kaan Boztug, Thomas Braschler, Sophie Bravo, Robbert Bredius, Philip Bright, Carolina Brito de Azevedo Amaral, Nicholas Brodszki, Grit Brodt, Allan Brolund, Pauline Brosselin, Bastian Brummel, Sonja Brun-Schmid, Jürgen Brunner, Roswitha Bruns, Christina Buchta, Dietke Buck, Aileen Bücker, Martina Bührlen, Stefan Burdach, Siobhan Burns, Janet Burton, Luis Caminal, Caterina Cancrini, Clementina Canessa, Nathan Cantoni, Brindusa Capilna, Fabiola Caracseghi, Isabel Caragol, Javier Carbone, Maria Carrabba, Latanya Chamberlain, Anita Chandra, Christophe Chantrain, Helen Chapel, Julie Chassot, Ronnie Chee, Matteo Chinello, Charu Chopra, Zita Chovancova, Martin Christmann, Krystyna Chrzanowska, Peter Ciznar, Karlien Claes, Carl Friedrich Classen, Martin Classen, Alexis-Virgil Cochino, Alison M. Condliffe, Selim Corbacioglu, Ana Isabel Cordeiro, Donatienne Cordier Wynar, Claire Core, Laurence Costes, Tanya Coulter, Virginie Courteille, Maria Cristina, Maria Cucuruz, Leonik Nel Dabrowska, Mandy Dähling, Mary Louise Daly, Claudia-Sabrina Daniel, Maria Giovanna Danieli, James Darroch, Graham Davies, Frans de Baets, Christiane De Boeck, Javier De Gracia Roldan, Narimene de Nadai, Iris de Schutter, Nathalie De Vergnes, Esther de Vries, Josine de Witte, Theo Debert, Corina Defila, Judith Deimel, Diane Delaplace, Rosa Maria Dellepiane, Nelli Dellert, Laura Delliera, Anita Delor, Ulrike Demel, John Dempster, M.M. den Os, Rosmarie Dengg, Sora Asfaw Desisa, Alexandra Desta, Drahomira Detkova, Maite Dewerchin, Romina Dieli Crimi, Dagmar Dilloo, Florentia Dimitriou, Jasmin Dinser, Nabila Dipani, Johannes Dirks, Anna-Maria Dittrich, Lylia Djermane, Yagmur Dogru, Figen Dogu Esin, Angelika Dombrowski, Michaela Döring, Camilla Heldbjerg Drabe, Susann Drerup, Barbara Drexel, G.J.A. Driessen, Gregor Dückers, Yasmine Dudoit, Andrea Duppenthaler, Georg Ebetsberger-Dachs, Mate Barnabas Ecser, Maartje Eekman, Rosanna Ehrat, Eva Eisl, Olov Ekwall, Rabab El Hawary, Sabine M. El-Helou, Aisha El-Marsafy, Sarina Elbe, Suzanne Elcombe, Alia Eldash, Youssif Alkady Radwa Salah Eldeen, P.M. Ellerbroek, Roland Elling, Jane Elliott, Angelika Engelhardt, Diana Ernst, Fügen Ersoy, Stefanie Esper, Isabel Esteves, Andrew Exley, Martin Faber, Giovanna Fabio, Gaby Fahrni, Laura Eva Faletti, Laura Eva Faletti, Claire-Michele Farber, João Farela Neves, Emilia Faria, Henriette Farkas, Evangelia Farmaki, Maria Faßhauer, Anders Fasth, Gerd Fätkenheuer, Stefanie Faustmann, Gisela Fecker, Conleth Feighery, Cornelia Feiterna-Sperling, Perez Eduardo Fernandez-Cruz, Cordeiro Ferreira Concalo, Alice Ferster, Tobias Feuchtinger, Oliver Feyen, Daniela Finke, Sigrid Fitter, Stefan Flaschberger, Lucia Fleckenstein, Dirk Föll, Adriano Fontana, Concetta Forino, Elisabeth Förster-Waldl, Nora Franchet, Dagmar Freitag, Urs P. Frey, Elisabeth Friedel, Wilhelm Friedrich, Barbara Frisch, Lukas Frischknecht, Stephanie Fritzemeyer, Alenka Gagro, Manfred Gahr, Eleonora Gambineri, Agnes Gamper, Franziska Gams, Astrid Ganzow, Nicolas Garcelon, Tomaz Garcez, Marina Garcia Prat, Ann Gardulf, Janine Garibay, Birgit Garwer, Jonathan Gathmann, Corinna Gebauer, Linda Geberzahn, Tilman Geikowski, Ulf Geisen, Christiane Gemander, Andrew R. Gennery, Marie Gerisch, Michael Gernert, Katrin Gerrer, Stev Gerschmann, Carolin Giannini, Juana Gil Herrera, Matthias Girndt, Ramona Girrbach, Hermann Girschick, Ioannis Gkougkourelas, Dominika Gładysz, Elisabeth Gnodtke, Vera Goda, Sarah Goddard, Daniela Goebel, Jean-Christophe Goffard, Sigune Goldacker, Eva Maria Gollowitsch, Manuella Gomes, Mark Gompels, Míriam González, Luis Ignacio Gonzalez Granado, Pavels Gordins, Katharina Gößling, Lisa Göschl, Lucy Gossens, Ewelina Gowin, Lea Graafen, Leo Graca, Brigita Gradauskiene (Sitkauskiene), Dagmar Graf, Norbert Graf, Elliot Grange, H. Anne Grashoff, Johann Greil, Sofia Grigoriadou, Helen Gronlund, Ute Groß-Wieltsch, Teresa Guerra, Kissy Guevara-Hoyer, Seny Gueye Mor, Noemie Guibert, Birgit Gülnur, Tayfun Güngör, Marina Guseva, David Guzman, Marcel Haag, Gabriele Haase, Henriette Haenicke, Filomeen Haerynck, Ines Hafsa, David Hagin, Michael Hallek, Gonca Hancioglu, Rupert Handgretinger, Leif G. Hanitsch, Susanne Hansen, Tetyana Hariyan, Thomas Harrer, Pia Hassunah, Maria Hatzistilianou, Fabian Hauck, Thomas Hauser, Margje H. Haverkamp, Grant Hayman, Paul Heath, Christian Hedrich, Maximilian Heeg, Michael Heike, Martin Heimbrodt, Sabine Heine, Ulrich Heininger, Christian Heinrich, Valerie Heinz, Andreas Heitger, Matthew Helbert, Arthur Helbling, Antje Hellige, Julya Hempel, Karen Henderson,

Downloaded from http://rupress.org/jhi/article-pdf/1/3/e20250007/1947361/jhi_20250007.pdf by guest on 17 July 2025

https://doi.org/10.70962/jhi.20250007

Jörg Henes, Philipp Henneke, Christian Hennig, Karin Henrichs, Martin Herbst, Walter Hermann, Manuel Hernandez, Anja Hernández, Edyta Heropolitanska, Richard Herriot, Friedrich Herrmann, Archana Herwadkar, Christoph Hess, Ursula Hess, Sebastian Hesse, Sonja Higgins, Anna Hilfanova, Sophie Hilpert, Chantal Hintze, Eva Hlaváčková, Isabel Hodl, Adna Hodzic, Miriam Hoernes, Christina Hoffmann, Andreas Holbro, Christiane Höllinger, Lisa Holtsch, Ursula Holzer, Dirk Holzinger, Manfred Hönig, Andrea Hönscheid, Julia Horn, Gerd Horneff, Claire Hoyoux, Nataliya Hristova, Kai Hübel, Angela Hübner, Christian Huemer, Aarnoud Huissoon, Brigitte Hülsmann, Patrick Hundsdörfer, Hans-Iko Huppertz, Kristina Huß, Sadia Hussain, Julien Husson, Tanja Hüttner-Foehlisch, Hanna Ijspeert, Aydan Ikinciogullari, Kökçü İlknur, Ninela Irga, Alexandra Jablonka, Karina Jahnz-Rozyk, Marcus Jakob, Donate Jakoby, Peter Jandus, Annette Jansson, Melanie Jaquet, Helene Jardefors, Edyta Jargulinska, Barbara Jauk, Milos Jesenak, Katharina Jilka, Stephen Jolles, Alison Jones, Regina Jones, Birgit Jonkman-Berk, Göran Jönsson, Hilary J. Joyce, Pricillia Juliana, Michael Kabesch, Leo Kager, Christian Kahlert, Petra Kaiser-Labusch, Ioannis Kakkas, Dirk Kamitz, Maria Kanariou, Lothar Kanz, Elif Karakoc-Aydiner, Boris Karanovic, Mutlu Kartal-Kaess, Elisabeth Käser, Terese L. Katzenstein, Hana Kayserova, Peter Kelleher, Tessa Kerre, Sara Sebnem Kilic, Martina Kirchner, Simone Kiwit, Ayca Kiykim, Jessica Klasen, Maja Klaudel-Dreszler, Ariane Klein, Christoph Klein, Andreas Klein-Franke, Ilona Kleine, Stefan Kleinert, Christian Klemann, Marion Klima, Robin Kobbe, Dilara Fatma Kocacik Uygun, Melanie Koch, Yvonne Kochler, Naschla Kohistani, Marina Kojic, Antonio Kolios, Uwe Kölsch, Sylwia Koltan, Irina Kondratenko, Christoph Königs, Julia Konoplyannikova, Peter Kopac, Jana Kopp, Dieter Körholz, Julia Körholz, Pauline Korte, Larysa Kostyuchenko, Ina Kötter, Sven Kracker, Pavlina Králícková, Christof Kramm, Philipp Kramme, Máté Krausz, Wolfhart Kreuz, Gergely Kriván, Gabriele Kropshofer, Renate Krüger, Olga Krystufkova, Maria Ktistaki, Jörn-Sven Kühl, Alexander Kühn, Taco W. Kuijpers, Wietse Kuis, Silke Kullmann, Andreas Kulozik, Dinakantha Kumararatne, Ria Kümmler, Andrea Kündgen, Magdalena Kurenko – Deptuch, Cosima Kuss Paula, Necil Kütükcüler, Cécile Lafoix-Mignot, Beate Lamers, Peter Lanbeck, Paul Landais, Sybille Landwehr-Kenzel, Vanessa Langemeyer, Thorsten Langer, Petra Lankisch, Nadia Lanz, Manrique de Lara, Eusebia Lara-Villacanas, Lisa Laubenthal, Hans-Jürgen Laws, Loic Le Mignot, Ronan Leahy, Jae-Yun Lee, Andrea Lehmann, Kai Lehmberg, Patricia Lehner, Hans Leibfrit, Leoni Leistner, Petra Lesch, Simon Leutner, Manolis Liatsis, Linda Libai Véghová, Johanna Liebel, Johannes G. Liese, Kotryna Linauskiene, Richard Linde, Martina Linßner, Conrad Ferdinand Lippert, Jiri Litzman, Pilar Llobet, Babacar Lo, Tariq Lodin, Jindrich Lokaj, David Longhino, Hilary Longhurst, Susana Lopes da Silva, Catharina Lorenzen, Vassilios Lougaris, Doris Löw, Annelie Lubatschofski, Mary Lucas, Verena Lutz-Wiegers, Sabine Maaß, Maria Elena Maccari, Anna Macura-Biegun, Vanessa Maerz, Paraskevi Maggina, Hannah Mahrenholz, Sarah Maier, Anne Malfroot, Laura Malinauskiene, Wilma Mannhardt-Laakmann, Ania Manson, Felicia Mantkowski, Petra Manzey, Carolina Marasco, Nufar Marcus-Mandelblit, Wolfgang Marg, Gasper Markelj, Laszlo Marodi, José Goncalo Marques, Laura Marques, Karin Marschall, Natalia Martínez, Rafael Martinez de la Ossa Saenz-Lopez, Inmaculada Martinez-Saguer, Baldassarre Martire, Antonio Marzollo, Refiloe Masekela, Katja Masjosthusmann, Tania Nicole Masmas, Nuria Matamoros, Jutta Mattern, Pearl Mau-Asam, Elizabeth McDermott, Nichole McIntosh, Karmen Mesko Meglic, Ruben Meijer, Andrea Meinhardt, Safa Meshaal, Yasmina Messaoud, Björn Meyer, Dirk Meyer-Olson, Romain Micol, Bozena Micoloc, Gudrun Mielke, Cinzia Milito, Siraj Misbah, Michael Mohr, Karina Mohrmann, Mostafa Moin, Luis Molinos, Jana Möller, Sarah Möller-Nehring, Henner Morbach, Viviana Moschese, Olga Moser, Despina Moshous, Radoslaw Motkowski, Jayashree Motwani, Nermeen Mouftah Galal, Carmen Müglich, Anna Mukhina, Eva Muller, Christiane Müller, Gabriele Müller, Hedi Müller, Ingo Müller, Thomas Müller, Zoe Müller, Ulf Müller-Ladner, Sarah Müller-Stöver, Esther Münstermann, Núria Murtra Garrell, Moritz Muschaweck, Miriam Mutert, Zohreh Nademi, Paru Naik, Andrea Näke, Andrea Martin Nalda, Gulnara Nasrullayeva, Nora Naumann-Bartsch, Verena Nemitz, Olaf Neth, Andreas Neubauer, Jennifer Neubert, Carla Neumann, Tim Niehues, Mara Niemuth, Chris Nieuwhof, Daniela Nolkemper, Sadia Noorani, Budde Adya Noorlander, Gundula Notheis, Sanam Amelie Nowatsh, Gaelle Obenga, Suheyla Ocak, Mehmet Oker, Peter Olbrich, Anna Olipra, Heymut Omran, Prasad Oommen, Jaroslava Orosova, Solveig Oskarsdottir, Hülya Özsahin, Malgorzata Pac, Jana Pachlopnik Schmid, Franco Pandolfi, Efimia Papadopoulou-Alataki, Anna Papatriantafillou-Schmieder, Alba Parra-Martinez, Olga Paschenko, Srdjan Pašić, Jarek Pasnik, Smita Patel, Martin Pavlík, Estela Paz Artal, Anouk Peeters, Sara Branco Pereira da Silva, Ruy Perez-Becker, Markus Perlhagen, Hans-Hartmut Peter, Michael Pfreundschuh, Pierre Philippet, Capucine Picard, Barbara Pietrucha, Leonora Pietzsch, Monica Piquer Gibert, Kerstin Pirolt, Alessandro Plebani, Daniel E. Pleguezuelo, Katrin Pollok, Helena Pommerening, Daniela Popihn, Aleksandra Poplonek, Marina Popp, Fulvio Porta, Jan Portegys, Klara Posfay-Barbe, Judith Potjewijd, Sebastian Poulheim, Seraina Prader, Petra Prämassing-Scherzer, Martina Prelog, Johan Prevot, Arthur Price, Timothy Price, Marijke Proesmans, Johan Provot, Isabella Quinti, Anna Raab, Anita Rack, Stefan Raffac, Eduardo Ramos Oviedo, Philippe Randrianomenjanahary, Anja Ranohavimparany, Maria Raptaki, Roonaka Rashidzadeh, Margit Rathwallner, Shereen Reda, Nahida Redouane, Frederico S. Regateiro, Janine Reichenbach, Bianca Reimers, Cornelia Reinhardt, Dirk Reinhardt, Anne Reinprecht, Tamara Reiß, Ismail Reisli, Eleonore Renner, Nima Rezaei, Alex Richter, Darko Richter, Nikolaus Peter Rieber, Nadja Rieckehr, Marion Riedel, Heidi Riescher, Johannes Rischewski, Nicole Ristl, Henrike Ritterbusch, Tanja Ritz, Francois Rivier, Jürgen K. Rockstroh, Joachim Roesler, Himatur Rofiah, Elizabeth Rogerson, Elisabeth Rolfes, Beate Roller, Yaryna Romanyshyn, Roberto Rondelli, Fien Roosens, Angela Rösen-Wolff, Valentina Rösler, Johannes Roth, Tobias Rothoeft, Agathe Roubertie, Gesa Rübsam, Abraham Rutgers, Paul Ryan, Gudrun Sach, Kambis Sadeghi, Ulrike Sahrbacher, Angelika Saidi, Özden Sanal Tezcan, Silvia Sanchez-Ramon, Juan Luis Santos, Ravishankar Sargur, Ihor Savchak, Sinisa Savic, Bernhard Schaaf, Marzena Schaefer, Christina Schäfe, Eva Scharbatke, Ellen Schatorje, Uwe Schauer, Carmen Scheibenbogen, Raphael Scheible, Clemens Scheinecker, Romana Schiller, Beatrice Schilling, Freimut Schilling, Steffi Schlieben, Thilo Schmalbach, Marc Thomas Schmalzing, Pirmin Schmid, Nadine Schmidt, Reinhold Ernst Schmidt, Monika Schmitz, Dominik Schneider, Dominik T. Schneider, Reinhard

Downloaded from http://rupress.org/jhi/article-pdf/1/3/e20250007/1947361/jhi_20250007.pdf by guest on 17 July 2025

Schneppenheim, Cathy Scholtes, E.H. Schölvinck, Stefan Schönberger, Stefan Schreiber, Rik Schrijvers, Andrea Schroll, Simone Schruhl, Johanna Schrum, Ralf Schubert, Sebastian Schuh, Ansgar Schulz, Claudia Schulz, Ilka Schulze, Hendrik Schulze-Koops, Ulf Schulze-Sturm, Eva-Maria Schumacher, Elvira Schürmann, Gesine Schürmann, Volker Schuster, Eva Schwaneck, Klaus Schwarz, Tobias Schwarz, Carolynne Schwarze-Zander, Lothar Schweigerer, Wolfgang Schwinger, Anna Sediva, Jörg Seebach, Reinhard Seger, Florian Segerer, Barbara Selle, Suranjith Seneviratne, Hatidje Shabanaj, Anna Shcherbina, Anne Siepelmeyer, Kathrin Siepermann, Anna Simon, Arne Simon, Katja Simon-Klingenstein, Marija Simonovic, Merlin Simsen-Baratault, Elena Sindram, Alla Skapenko, Maiken Skarke, Malgorzata Skomska-Pawliszak, Mary Slatter, Julie Smet, Ali Sobh, Bettina Sobik, Georgios Sogkas, Michael Sohm, Xavier Solanich-Moreno, Pere Soler Palacín, Franz Sollinger, Raz Somech, Anja Sonnenschein, Annarosa Soresina, Marijke Sornsakrin, Stavrieta Soura, Sabrina Spaccarotella, Guiseppe Spadaro, Monika Sparber-Sauer, Christof Specker, Carsten Speckmann, Lisa Speidel, Matthaios Speletas, Klaus-Daniel Stachel, Catherine Stadon, Aurélie Stanislas, Cynthia Stapornwongkul, Paulina Staus, Regina Steck, Cathal Steele, Herbert Steffin, Urs Steiner, Sandra Steinmann, Wim Stevens, Martina Stiefel, Sarah Stieger, Sophie Stiehler, Hermann Stimm, Silvia Stojanov, Matthias Stoll, Dominique Stoppa-Lyonnet, Tobias Strapatsas, Gabriele Strauß, Monika Streiter, Riet Strik-Albers, Gaby Strotmann, Héctor Suárez Casado, Jose Luis Subiza, Mikael Sundin, Birgit Süß, Fabienne Sutter, Anna Szaflarska, Monika Szemkus, Hannah Tamary, Sofia Tantou, Michael D. Tarzi, Helga Taschner, Ulf Tedgard, Carla Teixeira, RJM ten Berge, Klaus Tenbrock, Sabine Tester, Ilhan Tezcan, Sonja Thalguter, Julian Thalhammer, Katharina Thoma, Moira Thomas, Fabian Thomczyk, Vojtech Thon, Adrian Thrasher, Patricia Tierney, Nadine Tietsch, Alberto Tommasini, Beate Tönnes, Hans-Peter Tony, Maria Trachana, Carmen Trapp, Lourdes Tricas, Maria Conceicao Trindade Neves, Jordis Trischler, Hugo Ubieto, Anett Uelzen, Annette Uhlmann, Jan Ullrich, Kurt Ullrich, Ekrem Ünal, Gerhard Urbanski, Simon Urschel, Aleksandra Uszynska, Angelo Vacca, N.N. Vaganov, Daniel Vagedes, Stefanie Valicevic, Florence Vallelian, Rachel T. van Beem, Charlotte van Damme, Annick van de Ven, J. Merlijn van den Berg, Michiel van der Flier, J.T. van Dissel, P.M. van Hagen, J.M. van Montfrans, Geoffrey van Ogtrop, Jacqui van Rens, Christel A.M.P. van Riel, Annet van Royen-Kerkhof, G.Th.J. van Well, Antari Vasiliki, Sirje Velbri, Jo Vencken, Alessandro Ventura, François Vermeulen, Christiane Vermylen, Dorothee Viemann, Anja Viereck, Anna Villa, Jeroen Vincke, Lisa Vinnemeier-Laubenthal, Kim Duy Vo Thi, Kristina Vollbach, Alla Volokha, Horst von Bernuth, Philipp von Bismarck, Rebecca Voss, Sandra Voß, Yüksel Vural, Heike Wachuga, Norbert Wagner, Per Wagström, Matthias Wahle, Volker Wahn, Nadine Wapp, Klaus Warnatz, Monika Warneke, Adilia Warris, Jan-Christian Wasmuth, Jean-Blaise Wasserfallen, Angela Wawer, Manfred Weber, Corrie MR Weemaes, Julius Wehrle, Stephan Weidinger, Michael Weiß, Elisabeth Weißbarth-Riedel, Antje Werner, Sara Wessel, Marco Westkemper, Monika Wicher, Lutz Wickmann, Sabine Wiegert, Monique Wiehe, Katharina Wiehler, Lydia Wiesböck, Ewa Wiesik-Szewczyk, Anthony Williams, Beate Winkler, Christel Winkler, Martina Winkler, Melanie Winkler, Uwe Wintergerst, Lukas Wisgrill, Torsten Witte, Helmut Wittkowski, Barbara Wolf, Sandra Wölke, Christina Wolschner, Beata Wolska-Kusnierz, Philip Wood, Sarita Workman, Austen Worth, Michaela Wortmann, Walter Alfred Wuillemin, Nico M. Wulffraat, Katharina Wustrau, Chloé Wyndham-Thomas, Olcay Yegin, Alisan Yildiran, Denise Yilmaz, Patrick Young, Esra Yucel, Milica Zečević, Fred Zepp, Klaus Zetzsche, Rainald Zeuner, Stefan Zielen, Martina Zimmermann, Erić Želimir, Wadih Abou-Chahla, Nathalie Aladjidi, Corinne Armari-Alla, Vincent Barlogis, Sophie Bayart, Stéphane Blanche, Damien Bodet, Bernard Bonnotte, Raphaël Borie, Patrick Boutard, David Boutboul, Claire Briandet, Jean-Paul Brion, Jacques Brouard, Martin Castelle, Pascal Cathebras, Liana Carausu, Emilie Catherinot, Nathalie Cheikh, Morgane Cheminant, Thibault Comont, Louis-Jean Couderc, Pierre Cougoul, Anne Deville, Catherine Devoldere, Eric Dore, Fabienne Dulieu, Isabelle Durieu, Natacha Entz-Werle, Claire Fieschi, Lionel Galicier, Virginie Gandemer, Martine Gardembas, Clément Gourguechon, Bernard Grosbois, Aurélien Guffroy, Corinne Guitton, Gaëlle Guillerm, Mohamed Hamidou, Sophie Haro, Yves Hatchuel, Olivier Hermine, Cyrille Hoarau, Sébastien Humbert, Arnaud Jaccard, Jean-Philippe Jais, Sarah Jannier, Serge Jacquot, Roland Jaussaud, Pierre-Yves Jeandel, Eric Jeziorski, Kamila Kebaïli, Anne-Sophie Korganow, Olivier Lambotte, Fanny Lanternier, Claire Larroche, David Launay, Guillaume Le Guenno, Emmanuelle Le Moigne, Marc Lecuit, Guillaume Lefèvre, Jean-Daniel Lelièvre, Romain Levy, Valérie Li-Thiao-Te, Olivier Lortholary, Luminita Luca, Coralie Mallebranche, Marion Malphettes, Aude Marie-Cardine, Nicolas Martin-Silva, Agathe Masseau, Etienne Merlin, Frédéric Millot, Charline Miot, Floriane Mirgot, Nabila Moussouni, Luc Mouthon, Antoine Néel, Bénédicte Neven, Dalila Nouar, Raphaële Nove-Josserand, Marie Ouachée-Chardin, Anne Pagnier, Catherine Paillard, Marlène Pasquet, Isabelle Pellier, Antoinette Perlat, Christophe Piguet, Dominique Plantaz, Sophie Rivière, Loïc Raffray, Pascal Roblot, Pierre-Simon Rohrlich, Bruno Royer, Valéry Salle, Hélène Salvator, Françoise Sarrot-Reynauld, Amélie Servettaz, Jean-Louis Stephan, Nicolas Schleinitz, Felipe Suarez, Laure Swiader, Sophie Taque, Caroline Thomas, Olivier Tournilhac, Caroline Thumerelle, Jean-Pierre Vannier, Jean-François Viallard.

Downloaded from http://rupress.org/jhi/article-pdf/1/3/e20250007/1947361/jhi_20250007.pdf by guest on 17 July 2025
